# Current and future potential distributions of three *Dracaena* Vand. ex L. species under two contrasting climate change scenarios in Africa

**DOI:** 10.1002/ece3.5251

**Published:** 2019-06-11

**Authors:** Paweł Bogawski, Theo Damen, Maciej M. Nowak, Katarzyna Pędziwiatr, Paul Wilkin, Geoffrey Mwachala, Joanna Pierzchalska, Justyna Wiland‐Szymańska

**Affiliations:** ^1^ Laboratory of Biological Spatial Information, Faculty of Biology Adam Mickiewicz University Poznań Poland; ^2^ Independent Scholar Wageningen The Netherlands; ^3^ Royal Botanic Gardens, Kew Richmond UK; ^4^ East African Herbarium National Museums of Kenya Nairobi Kenya; ^5^ Department of Plant Taxonomy, Faculty of Biology Adam Mickiewicz University Poznań Poland

**Keywords:** *Dracaena afromontana* Mildbr., *Dracaena camerooniana* Baker, *Dracaena surculosa* Lindl., ecological niche modeling, forest undergrowth plants, global warming, Maxent model, rainforest, shade‐demanding species

## Abstract

Forest undergrowth plants are tightly connected with the shady and humid conditions that occur under the canopy of tropical forests. However, projected climatic changes, such as decreasing precipitation and increasing temperature, negatively affect understory environments by promoting light‐demanding and drought‐tolerant species. Therefore, we aimed to quantify the influence of climate change on the spatial distribution of three selected forest undergrowth plants, *Dracaena* Vand. ex L. species, *D. afromontana* Mildbr., *D. camerooniana* Baker, and *D. surculosa* Lindl., simultaneously creating the most comprehensive location database for these species to date.

A total of 1,223 herbarium records originating from tropical Africa and derived from 93 herbarium collections worldwide have been gathered, validated, and entered into a database. Species‐specific Maxent species distribution models (SDMs) based on 11 bioclimatic variables from the WorldClim database were developed for the species. HadGEM2‐ES projections of bioclimatic variables in two contrasting representative concentration pathways (RCPs), RCP2.6 and RCP8.5, were used to quantify the changes in future potential species distribution.

*D. afromontana* is mostly sensitive to temperature in the wettest month, and its potential geographical range is predicted to decrease (up to −63.7% at RCP8.5). Optimum conditions for *D. camerooniana* are low diurnal temperature range (6–8°C) and precipitation in the wettest season exceeding 750 mm. The extent of this species will also decrease, but not as drastically as that of *D. afromontana*. *D. surculosa* prefers high precipitation in the coldest months. Its potential habitat area is predicted to increase in the future and to expand toward the east.

This study developed SDMs and estimated current and future (year 2050) potential distributions of the forest undergrowth *Dracaena* species. *D. afromontana*, naturally associated with mountainous plant communities, was the most sensitive to predicted climate warming. In contrast, *D. surculosa* was predicted to extend its geographical range, regardless of the climate change scenario.

## 1 INTRODUCTION

The genus *Dracaena* Vand. ex L. belongs to the family Asparagaceae (APG III, 2009). It is considered to be monophyletic based on molecular studies (Lu & Morden, 2010) and comprises approximately 116 species (Govaerts et al. 2018). *Dracaena* is distributed in subtropical and tropical regions of the world. Most species are found in Africa, Madagascar, and Asia, and the remainder are found in Socotra, the Mediterranean region, Macaronesia, Central America, Cuba, Micronesia, Australia, and the Pacific Islands (Bos, 1998; Marrero, Almeida, & González‐Martín, 1998; Staples & Herbst, 2005). Approximately 63 species of *Dracaena* occur in Africa (incl. Madagascar) (Damen, Burg, Wiland‐Szymańska, & Sosef, 2018). Three of them belong to a group of dragon trees found in semiarid and subtropical climates (Marrero et al., 1998). The rest are connected with humid habitats (Bos, 1984; Pierzchalska, Nowak, Wilkin, Mwachala, & Wiland‐Szymańska, 2014).

The species of the genus *Dracaena* are among the most important ornamental plants in the world due to their various leaf shapes and colorations as well as their resistance to indoor conditions (Bos, 1984; Singh & Dadlani, 2000). Some species of *Dracaena* produce chemicals valued in medicine and cosmetology, as well as in traditional healing. The best examples are *Dracaena draco* L., *D. cinnabari* Balf. f., and *D. ombet* Heuglin ex Kotschy and Peyr., which produce a resin called “dragon's blood” (Elnoby, Moustafa, & Mansour, 2017; Jura‐Morawiec & Tulik, 2016), as well as *D. mannii* Baker and *D. arborea* (Wild.) Link (Okunji, Iwu, Jackson, & Tally, 1996). *Dracaena* species also play a role as markers of borders and other socially important sites in Africa (Sheridan, 2008). Spontaneously regenerating *D. afromontana* is utilized by farmers as a plant that provides ecosystem services on coffee plantations in Rwanda (Smith, Gassner, Agaba, Nansamba, & Sinclair, 2018). Fruits of *Dracaena* are forage for birds and mammals (González‐Castro, Pérez‐Pérez, Romero, & Nogales, 2019). In spite of its importance, the taxonomy and distribution of the genus *Dracaena* are not fully known, and recent works stress the need for further investigations of this genus (Damen et al., 2018; Pierzchalska et al., 2014). The habitat preferences of African *Dracaena* species have not been studied except for those of *D. ombet* (Robiansyah & Hajar, 2017). However, the difficulty of performing a continental‐scale, systematic botanical study in Africa (e.g., due to staff, funding and accessibility limitations) negatively impacts our understanding of the current distribution of *Dracaena* species on this continent. Increasing anthropogenic impact (e.g., cultivating ornamental plants) also prevents a reliable assessment of the current distribution of plants, especially those connected with fragile and overexploited habitats, for example, the equatorial forest (Ahmed & Mlay, 1998). The scarcity of botanical data acquired during planned botanical surveys can be overcome by historical herbarium materials that have been collected over decades. Gathering records from numerous herbaria worldwide may shed light on the current and future potential distribution of plant species when applying specially designed mathematical methods (Crawford & Hoagland, 2009; Elith & Leathwick, 2007).

Global climate variability is recognized as one of the main causes of changes in the spatial distribution of plants (Costion et al., 2015; Kelly & Goulden, 2008). One of the most susceptible ecosystems is the tropical forest, broadly defined as an area with tree canopy >5 m tall covering at least 10% of more than 0.5 ha surface and located between the tropics of Cancer and Capricorn (FAO, 2001). Tropical forests are global hotspots of biodiversity and significant modulators of climate change but are also among the most vulnerable ecosystems worldwide. Among them are rainforests or equatorial forests which are relatively dense, tall, and evergreen broadleaf forests with a high number of tree species growing in a moist environment (Lewis, 2006; Ter Steege et al., 2003). In recent years, these ecosystems have been decreasing due to logging and mining activities that lead to further land degradation and land use change over time (Laurance & Cochrane, 2001). However, even the apparently intact forest areas have been altered, especially in their physiology and ecology. A “CO_2_ fertilization” hypothesis suggests that the productivity and growth rate of tropical forests increase due to the higher amount of CO_2_ (originating from the combustion of fossil fuels) available for photosynthesis (Lloyd & Farquhar, 2008). It has been found that the dry biomass of trees in tropical rainforests increased by approximately 1 Mg ha^−1^ year^−1^ in last 20 years of the 20th century (Baker et al., 2004). One of the effects of this biomass increase will be a faster plant life cycle, a higher tree mortality rate, and consequently a more frequent occurrence of canopy gaps. This, in turn, will favor light‐demanding species and would be a limiting factor for shade‐demanding species such as forest undergrowth plants (Lewis, 2006; Lewis, Malhi, & Phillips, 2004). Therefore, there is a risk of the decline in forest undergrowth plant populations, even when located in intact rainforests far from direct human impacts. In this study, we investigated three *Dracaena* species that belong to this group of plants. They can serve as a proxy for other shade‐tolerant understory species.

The “CO_2_ fertilization” hypothesis is still broadly discussed worldwide, as there is also evidence that the aboveground net primary productivity in equatorial forests does not increase, despite the distinct increase in CO_2_ concentration in recent years (Clark, Clark, & Oberbauer, 2013). This may be an effect of recently observed drying, especially in western equatorial Africa (James, Washington, & Rowell, 2013). Additionally, long‐term satellite multisensor measurements of the vegetation conditions in equatorial forests show that forest greenness is declining in most parts of the African equatorial forest (Zhou et al., 2014). This photosynthetic capacity loss can be attributed to the drying tendency, which, consequently, may lead to modification of the forest species composition by favoring drought‐tolerant species (Fauset et al., 2012; Lewis, 2006). In this case, forest undergrowth plants that are typically adjusted to shady, wet environments will be more endangered. Therefore, regardless of whether the future conditions of tropical forests follow the trend of the “CO_2_ fertilization” hypothesis, the number and strength of threats to forest understory plants seem to be increasing more rapidly than for other ecological groups of plants in equatorial forests.

Therefore, the potential ranges of three *Dracaena* species growing as understory plants in equatorial forests were modeled in this study. A general purpose of species distribution modeling (SDM) is to estimate the potential geographical distribution of species based on the environmental conditions recorded at species record locations. For presence–absence data, it is possible to apply traditional ecological modeling techniques, such as GLM (generalized linear models), GAM (general additive models), and others (Franklin & Miller, 2009). Presence–absence data could be collected in a systematic, planned botanical study, which is frequently difficult due to time, funding, and staff limitations and to political situations (Elith et al., 2006). In contrast, presence‐only data are much easier to obtain but are more difficult to reliably use in SDM (Fithian & Hastie, 2013). To account for the frequent lack of the absence data, so‐called pseudo‐absence or background data are usually simulated, for example, using a Maxent model that is particularly designed for presence–background data (Phillips, Anderson, & Schapire, 2006; Phillips & Dudik, 2008). A Maxent model is able to predict not only the current species occurrence probability but also the probability in past and future climates (Febbraro et al., 2017; Kukwa & Kolanowska, 2016). Recently, the future climate change scenario generation process has been redesigned from a sequential to a parallel approach. The latter approach starts with the target radiative forcing in the year 2,100 and assumes that different combinations of affecting factors (e.g., policy, economy, land use changes) may contribute to the same target level. There are four main representative concentration pathways (RCPs) that generally reflect radiative forcing continuously rising (RCP 8.5), stabilizing (RCP 6.0 and RCP 4.5) or reaching a peak, and then declining (RCP 2.6) (Moss et al., 2010).

Considering the previously stated need to assess the influence of climate change on a vulnerable ecological group of plants (forest undergrowth species), we specified the following hypothesis for this study: As a representation of forest undergrowth plants, three selected *Dracaena* species will be dramatically affected by climate change‐induced loss of potential habitats, and therefore, a large proportion of the current potential range will be eliminated in the near future. To assess the future distribution patterns of the species in question, we aimed to (a) create a database with validated species occurrence records for the three *Dracaena* species in question, (b) identify the most important climate variables affecting the distribution of these *Dracaena* species, (c) determine the optimum climatic conditions for these *Dracaena* species, (d) estimate and discuss the current potential geographic range of these *Dracaena* species in comparison with the collected records, and (e) show future changes in the ranges of these *Dracaena* species according to two contrasting climate warming scenarios.

## 2 MATERIALS AND METHODS

### 2.1 *Dracaena* spp. occurrence records

**Figure 1:**
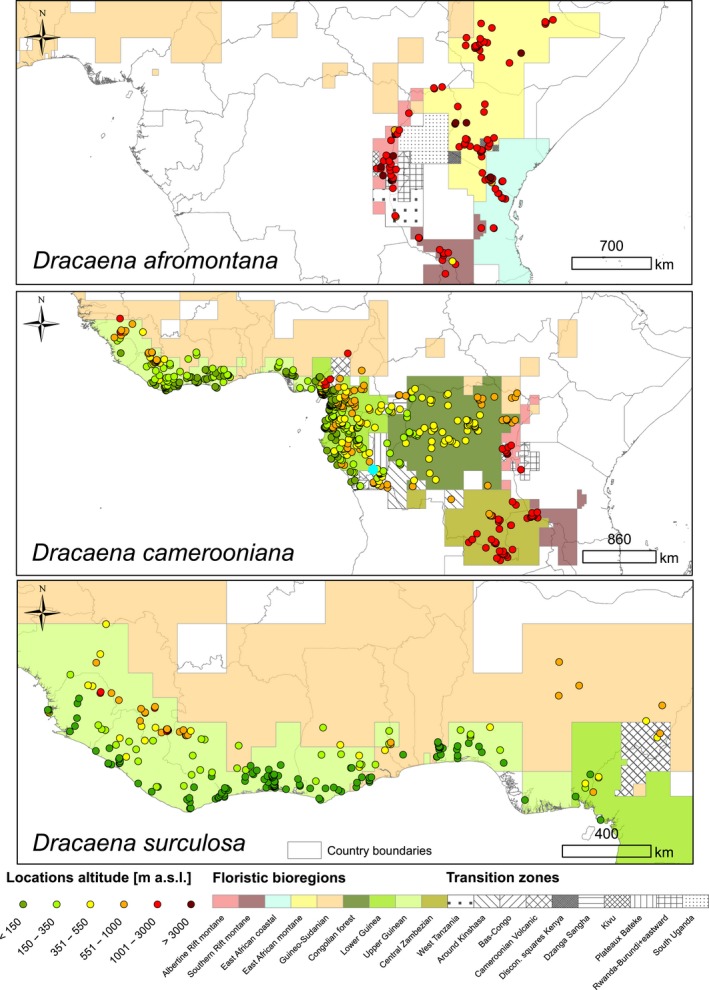
Location and altitude of presence records of three *Dracaena* species on the background of African floristic bioregions (Droissart et al., 2018)

For our study, we chose three species of *Dracaena* characterized by different spatial distributions: *D. afromontana* Mildbr., *D. camerooniana* Baker, and *D. surculosa* Lindl. *D. afromontana* is a 2–12 m high shrub or tree. It grows in Eastern and Central Africa (Figure 1) (Kelbessa, Kalema, & Crook, 2013). *Dracaena camerooniana* is a branched shrub with cane‐like shoots, 0.3–8 m in height, growing in Central and West Africa (Figure 1) (Damen et al., 2018). Both species are listed on the IUCN Red List as species of Least Concern (Crook, 2013; Kelbessa et al., 2013). *Dracaena surculosa* is also a branched shrub, 1–8 m in height naturally, that grows in West Africa (Figure 1) and is widely cultivated as an ornamental plant (Bos, 1984). More detailed information about the species studied can be found in Mwachala (2005).

Occurrence records originated from 93 herbaria around the world (a complete list in Appendix S1; abbreviations according to Thiers, 2018). We only took records of specimens that we had seen and that have an exact specified location in the wild (coordinates available). Finally, we validated the identification of 1,223 herbarium records: 161 *D. afromontana*, 771 *D. camerooniana,* and 291 *D. surculosa* (see Appendix S2). Some of the records were collected in the same location or close to each other (within a distance of 30 arc sec—approximately 1 km^2^ at the equator—the spatial resolution of bioclimatic variables: see section Environmental variables), which could introduce potential bias resulting from haphazard sampling (Syfert, Smith, & Coomes, 2013). To decrease the spatial autocorrelation, we removed all locations but one, attributed to one grid cell of the bioclimatic layers. This procedure allowed us to prevent the analysis from overweighting locations situated in easy‐to‐access, well‐surveyed areas (Newbold, 2010). Therefore, for modeling purposes, in the end, we used 920 independent locations (presence‐only data), with 122 records for *D. afromontana*, 588 for *D. camerooniana,* and 210 for *D. surculosa* (Figure 1). Altitudinal data used in this study were retrieved from GTOPO30, a global digital elevation model with a spatial resolution of 30 arc sec (the same as the bioclimatic data in this study, see below) and available at the U.S. Geological Survey's Center for Earth Resources Observation and Science (EROS). To ensure the uniformity of information for analysis, we did not use altitudinal data from herbarium labels.

### 2.2 Species distribution modeling

Maxent, a machine learning technique using a maximum entropy approach, has been used in this study. It generally means that of the different possible probability distribution functions describing and explaining the data, the most appropriate one is the distribution with maximal entropy within a set of constraints (Elith et al., 2011; Jaines, 1957). Maxent model results are presented in a readily interpretable logistic format that indicates areas with higher and lower probabilities of species occurrence. Maxent is considered to have comparable or better quality than other models of this type applied to presence–background data in SDM (Elith et al., 2006). We used the Maxent software, version 3.3.3k (Phillips et al., 2006; Phillips & Dudik, 2008) with the default parameters because they have been validated over many species and environmental variables, various sample sizes, and biases (Elith et al., 2011). The convergence threshold, maximum number of iterations, maximum number of background points for creating background data, and prevalence were set to 10^−5^, 5 × 10^2^, 10^4^ and 5 × 10^−1^, respectively.

The goodness‐of‐fit of the SDM models calibrated in this study was tested using the approach of a receiver operating characteristic (ROC) curve that shows the interdependence between true positive (sensitivity) and false positive (1‐specificity) rates of presence/background events of the observed/predicted data. The area under the ROC curve (AUC) was used as a measure for model accuracy. The AUC provides a simple numerical value (varying from 0.5—no predictive power—to 1—perfect discrimination) that quantifies the location of the ROC curve to a diagonal that describes a random model (Hanley & McNeil, 1982). The final testing AUC value for a particular species distribution model was obtained by 10‐fold cross‐validation (Christensen, 2002). The testing AUC values were justified according to the traditional classification of the model performance (Swets, 1988): poor (AUC = 0.5–0.6), fair (0.6–0.7), good (0.7–0.8), very good (0.8–0.9), and excellent (0.9–1).

### 2.3 Environmental variables

To define the ecological requirements and niches of the three *Dracaena* species, we used the bioclimatic variables from the Worldclim database v.1.4. (Hijmans, Cameron, Parra, Jones, & Jarvis, 2005, http://www.worldclim.org/) with a 30 arc‐sec spatial resolution. A complete list of the 19 explanatory variables was reduced to 11 bioclimatic variables (Table 1). The 8 redundant variables were removed based on the results of a pairwise Pearson correlation test incorporated in ENMTools (Warren, Glor, & Turelli, 2010) over the study area (Table S3.1 in Appendix S3). Only variables with <0.75 correlation proceeded to further analysis (as described in Ponce‐Reyes et al., 2017). We also reduced the number of variables by not considering altitude and other nonclimatic variables because adding such variables typically does not increase the AUC values (Vedel‐Sørensen, Tovaranonte, Bøcher, Balslev, & Barfod, 2013). Moreover, the additional information carried by such variables as elevation or aspect frequently overlaps with the information stored in climatic variables (Apaydin, Anli, & Ozturk, 2011; Shi, Paull, & Rayburg, 2016). Some authors indicate that the use of only climatic parameters as explanatory variables in SDM can be problematic (e.g., Wiens, Stralberg, Jongsomjit, Howell, & Snyder, 2009) because land use and land cover are very important factors that determine the species distribution. However, this kind of limitation particularly involves studies on smaller spatial scales (Pearson & Dawson, 2003). In contrast, the present study area comprises a large part of the African continent, and the herbarium records came from the entire known range of the selected *Dracaena* species, which improves the scientific value of the species distribution modeling (Cuyckens, Christie, Domic, Malizia, & Renison, 2016). Thus, we decided to focus on carefully selected bioclimatic variables only.

**Table 1:** Description of bioclimatic variables used in this study and retrieved from Worldclim database (Hijmans et al., 2005)

Variable description	Abbreviation
Mean Diurnal Temperature Range (difference between mean monthly maximum and minimum temperature)	Temp_range
Isothermality—the relationship between Mean Diurnal Temperature Range and Temperature Annual Range (difference between temp_max and temp_min)	Isotherm
Maximum temperature of the warmest month	Temp_max
Minimum temperature of the coldest month	Temp_min
Mean temperature of the wettest quarter	Temp_wet
Mean temperature of the driest quarter	Temp_dry
Precipitation seasonality—variation coefficient	Prec_var
Precipitation total of wettest quarter	Prec_wet
Precipitation total of driest quarter	Prec_dry
Precipitation total of the warmest quarter	Prec_warm
Precipitation total of the coldest quarter	Prec_cold

A relative contribution of variables to the overall Maxent model performance has been checked to identify the factors most likely limiting the occurrence of the *Dracaena* species studied. Two measures of the variable importance were (a) the permutation importance of a bioclimatic variable and (b) jackknife resampling analysis showing AUC with only a particular variable and without this variable. The values of the three variables with the highest contribution (most important) per species were plotted against the probability of occurrence, resulting in partial dependence plots.

### 2.4 Climate change scenarios, climate model

From four main existing representative concentration pathways (RCPs), we selected the best and the worst scenarios, called RCP 2.6 and RCP 8.5, respectively (Moss et al., 2010). By considering two opposite climate change scenarios, we were able to capture the possible variability (maximum–minimum interval) in future distribution changes of the three species of *Dracaena* studied.

To predict the future potential distributions of the three *Dracaena* species, we used projections of the Worldclim bioclimatic variables (Hijmans et al., 2005) calculated from future climate projections within the framework of the Coupled Model Intercomparison Project Phase 5 (CMIP5) (Taylor, Stouffer, & Meehl, 2012). The HadGEM2‐ES model (Collins et al., 2011) was selected for the present study based on the recommendation of this model to predict future temperature and precipitation changes in Africa (Dike et al., 2015). Other studies, such as that of Brands, Herrera, Fernandez, and Gutierrez (2013), confirmed that HadGEM2‐ES outperforms other models gathered in CMIP5 when compared for Africa.

### 2.5 Environmental niche mapping

The current and future potential distribution of the three *Dracaena* species was mapped using the maximum sensitivity + specificity logistic threshold (max SSS). This parameter is based on maximizing the sum of sensitivity and specificity and is recommended for use to binarize the occurrence probabilities (Liu, White, & Newell, 2013 and references therein). Gathering all the Maxent model iterations, we obtained a statistical distribution of the selected threshold for training and test datasets for each species (Figure S3.1 in Appendix S3). Finally, we mapped a median current potential distribution (current potential range), minimum and maximum possible range, and 1st and 3rd quartile range based on max SSS values. For future climate change scenarios, we mapped the median and minimum future range over the background of the current potential range. In addition, we calculated the current and future potential range area attributed to a particular country. Comparing this to the number of herbarium records per country may serve as a proxy for the completeness of distribution studies of selected *Dracaena* species in a country. Both the initial number of records and the final selection of unique locations are provided in the Supporting Information (see Tables S3.1, S3.2, S3.3. in Appendix S3). To provide more details on the future potential area of *Dracaena* species, we calculated future surface area changes based on two different climate warming scenarios.

## 3 RESULTS

### 3.1 Importance of the environmental variables

**Figure 2:**
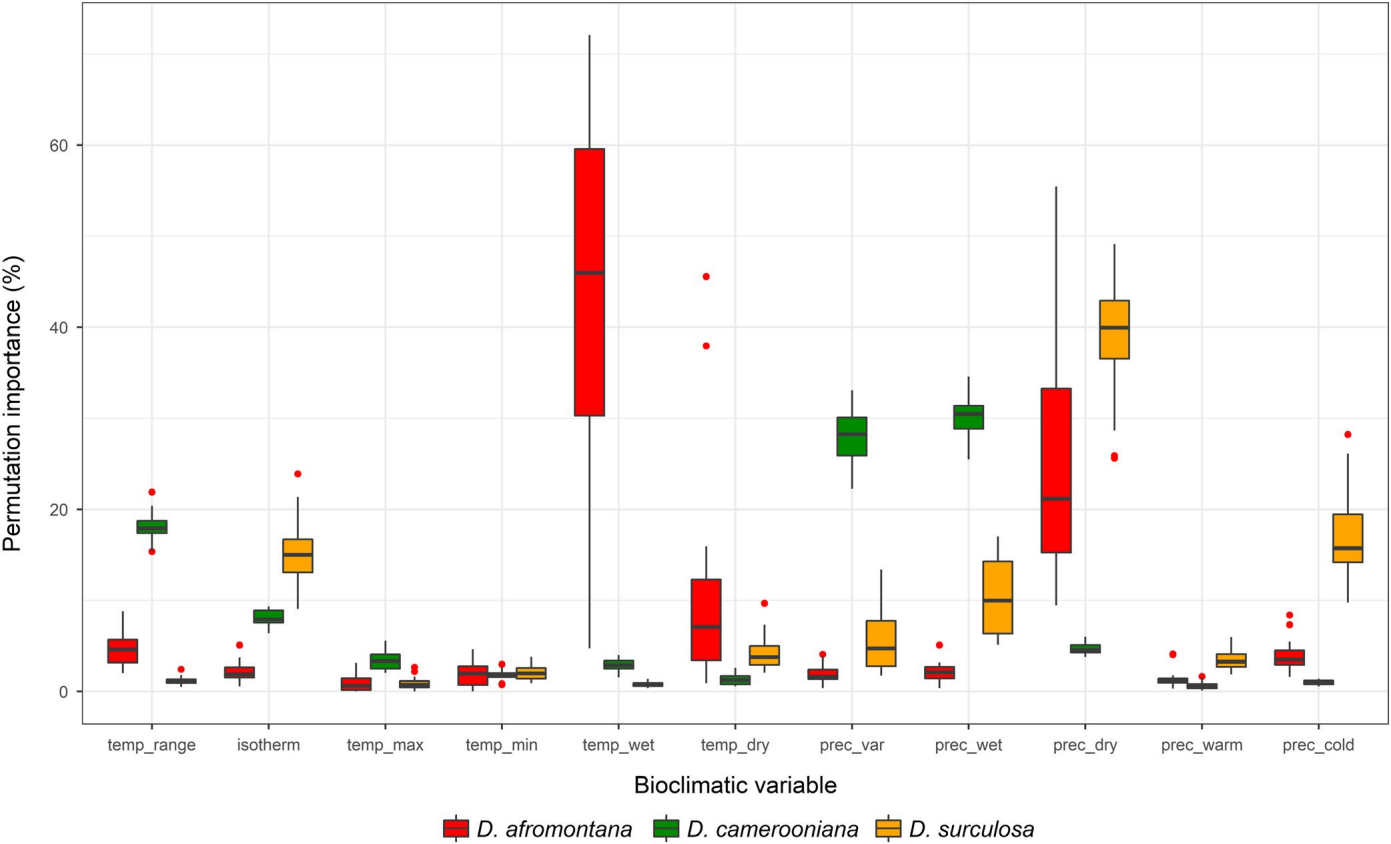
Relative contribution (permutation importance) of environmental variables to the final species distribution models for *Dracaena* species. Boxplot: central value—median, upper/lower hinges—1st and 3rd quartile, whiskers—extreme values

**Figure 3:**
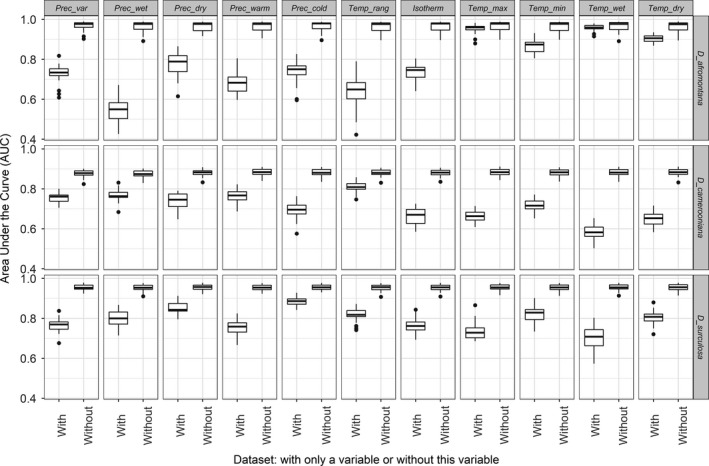
AUC values obtained at jackknife analysis for all the 11 bioclimatic variables used. AUC was calculated for partial models with only a particular variable and without this variable

For *D. afromontana*, temp_wet (46.0%) and prec_dry (21.2%) contributed most to the model performance (Figure 2). Temp_wet also turned out to be the most important variable when testing the AUC for individual variables (AUC = 0.96) (Figure 3). In *D. camerooniana*, three variables contributed at least 15%: prec_wet (30.5%), prec_var (28.3%), and temp_range (17.9%) (Figure 2). Using individual variables, temp_range resulted in an AUC higher than 0.8, and precipitation variables reached maximal AUC = 0.735 (prec_wet) (Figure 3). In *D. surculosa*, the three following variables were important: prec_dry (40.0%), prec_cold (15.7%), and isotherm (15.0%) (Figure 2). This was reflected in the AUC analysis, where prec_cold and prec_dry reached AUC = 0.887 and AUC = 0.845, respectively (Figure 3).

### 3.2 Potential optimum conditions

**Figure 4:**
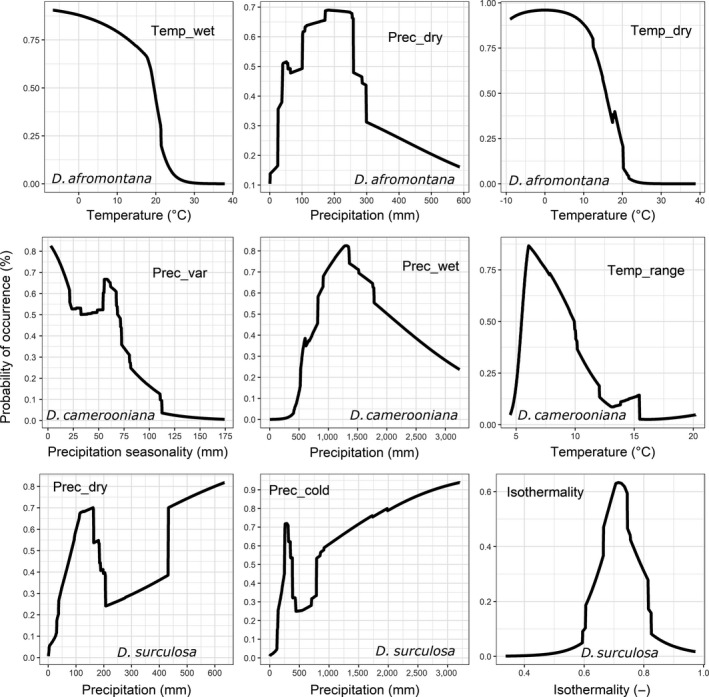
Partial dependence curves showing the response of *Dracaena* species to three variables mostly responsible for their spatial distribution (assuming that remaining variables are constant). The higher the probability of occurrence (vertical axis), the more suitable conditions for particular *Dracaena* species

The occurrence of *D. afromontana* was directly related to temp_wet and temp_dry. Occurrence probability sharply increased when these two variables were lower than 20°C and 15°C, respectively. In turn, the occurrence probability of *D. camerooniana* distinctly increased when prec_wet was >750 mm, prec_var was <70 mm, and temp_range was 6–8°C. In *D. surculosa*, the optimum conditions occurred when prec_cold exceeded 1,300 mm and prec_dry exceeded 430 mm (Figure 4).

### 3.3 Current potential distribution

*Dracaena afromontana* records were located mainly at higher altitudes in East Africa in the following floristic bioregions (Droissart et al., 2018): 2—East African montane, 5—Albertine Rift montane, and 9—Southern Rift montane. *D. camerooniana* occupies a wide area of Central Africa surrounding the Gulf of Guinea, concentrated mainly on its Eastern bank and in the Congo Basin, in the following bioregions: 3—Guineo‐Congolian, 5—Albertine Rift montane, and 8—Central Zambezian and transition zones. Numerous *D. camerooniana* records also originated from the areas adjacent to the north coast of the Gulf of Guinea that were sympatric to the *D. surculosa* locations (Figure 1), which is restricted to the 3—Guineo‐Congolian (including 3a—Upper Guinea and 3b—Lower Guinea) and 1—Guineo‐Sudanian bioregions.

**Figure 5:**
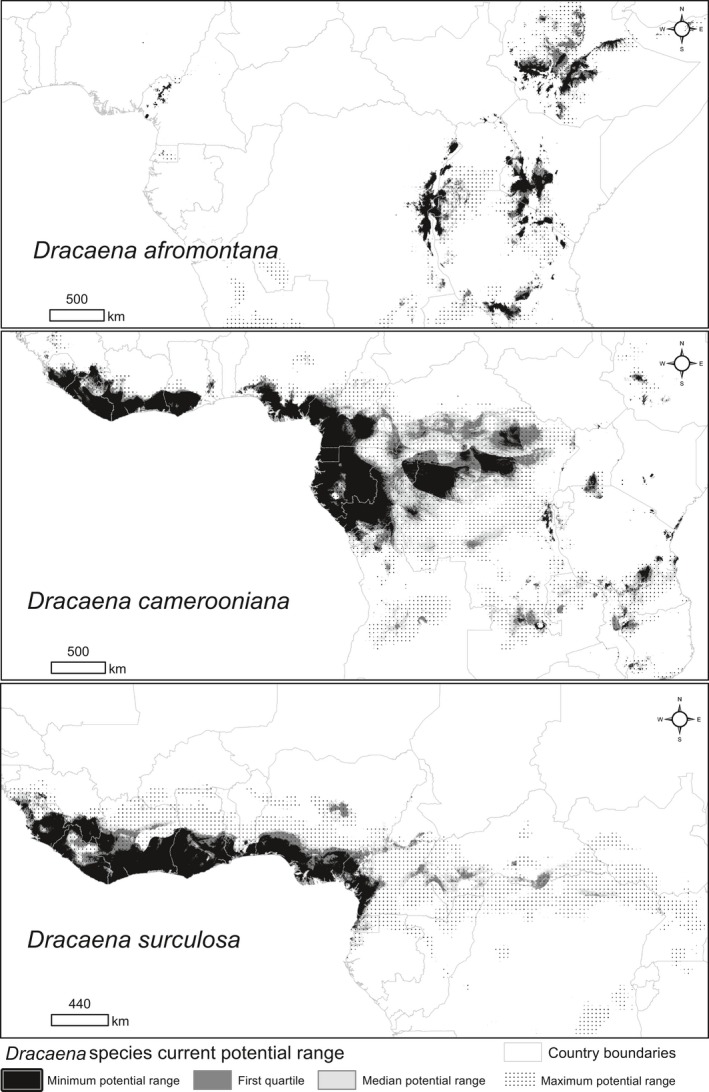
Current potential distribution of three *Dracaena* species in Africa. Current potential range of a particular species is attributed to median potential range. Maximum and minimum potential ranges were shown on the basis of minimum and maximum (respectively) values of max SSS logistic threshold obtained during the fitting of the models

Species distribution models developed in this study have been classified as excellent (*D. afromontana* and *D. surculosa*) or very good (*D. camerooniana*), reaching mean testing AUCs of 0.966, 0.955, and 0.883, respectively (Table 2). The entire modeled *D. afromontana* current potential range (573,400 km^2^) is located in the mountainous parts of nine African countries, with Ethiopia, Tanzania, Kenya, and DR Congo occupying 36.1%, 18.9%, 15.9%, and 11.4% of the current potential range, respectively. Most records were located in Tanzania (42) and Ethiopia (35). However, the highest record density (locations per 10,000 km^2^ of the country) was calculated for Burundi (5.14) (Table 3, Figure 5, Table S3.1 in Appendix S3).

**Table 2:** Validation scores of the three *Dracaena* species final distribution models

Species name	Training AUC			Testing AUC		
	Min	Mean ± *SD*	Max	Min	Mean ± *SD*	Max
*D. afromontana*	0.978	0.981 ± 0.002	0.984	0.901	0.966 ± 0.03	0.989
*D. camerooniana*	0.899	0.902 ± 0.002	0.906	0.837	0.883 ± 0.02	0.911
*D. surculosa*	0.965	0.967 ± 0.001	0.969	0.916	0.955 ± 0.02	0.978

Abbreviation: AUC, area under the receiver operation characteristic curve.

**Table 3:** Changes in three *Dracaena* sp. potential coverage until 2050 according to two contrasting climate change scenarios

Species	Statistical distribution of a potential range^a^	Current potential coverage (×1,000 km^2^)	Future potential coverage—climate change scenario			
			RCP2.6		RCP8.5	
			Area (×1,000 km^2^)	Relative change (%)	Area (×1,000 km^2^)	Relative change (%)
*D. afromontana*	Median range	573.4	250.1	−56.3	166.5	−63.7
	Minimum range	209.1	107.0	−48.8	76.0	−70.9
	Maximum range	1,531.0	599.1	−60.9	336.9	−78.0
*D. camerooniana*	Median range	2,698.5	1,954.6	−27.6	1,066.0	−60.5
	Minimum range	1,160.0	1,057.6	−8.8	539.2	−53.5
	Maximum range	4,450.7	3,102.0	−30.3	1,958.1	−56.0
*D. surculosa*	Median range	1,028.4	1,486.6	+44.6	1,763.1	+71.5
	Minimum range	578.9	820.7	+41.8	853.4	+47.4
	Maximum range	2,886.3	4,150.7	+43.8	4,853.8	+68.2

a Median range—potential distribution area calculated using median max SSS logistic threshold; Minimum range—potential distribution area calculated using maximum value of max SSS logistic threshold; Maximum range—potential distribution area calculated using minimum value of max SSS logistic threshold.

The current potential range of *D. camerooniana* covered 2,698,500 km^2^, mainly in the following countries: DR Congo (35.6% of current potential range), Congo (11.4%), Cameroon (9.6%), and Gabon (9.5%). Cameroon (185), DR Congo (179), and Gabon (129) had the most record locations in their territory, but the highest record density was noted in Equatorial Guinea (7.46 per 10,000 km^2^ of country area) (Table 3, Figure 5, Table S3.2 in Appendix S3).

The estimated *D. surculosa* current potential range covered 1,028,400 km^2^, primarily in Ivory Coast (18.8%), Nigeria (18,8%), Ghana (12,3%), and Cameroon (11.8%). Most herbarium records originated from Ivory Coast, and this country also had the highest record density (4.21 per 10,000 km^2^ of country area) (Table 3, Figure 5, Table S3.3 in Appendix S3).

### 3.4 Future potential distribution

**Figure 6:**
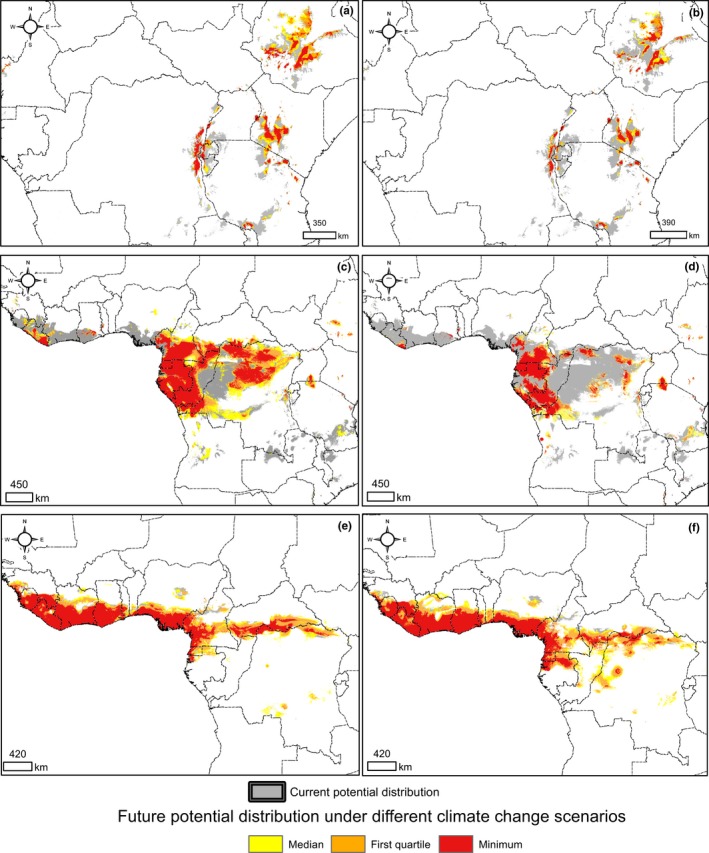
Future potential distribution of *Dracaena* species. Under two contrasting climate change scenarios (representative concentration pathways, RCPs), RCP 2.6 (left column) and RCP 8.5 (right column): (a and b) *Dracaena afromontana*; (c and d) *Dracaena camerooniana*; (e and f) *Dracaena surculosa*

Regardless of the climate change scenario, the area potentially occupied by *D. afromontana* and *D. camerooniana* is predicted to be markedly reduced by 2050 (Figure 6a–d). The former species seems to be even less resistant to climate warming than the latter; its potential distribution may be reduced by 56.3% in the RCP 2.6 scenario and 63.7% in the RCP 8.5 scenario (Table 3). Considering individual countries, the largest loss of *D. afromontana* potential range in the future involves Tanzania, where the range area will decrease dramatically regardless of the climate change scenario (at least 75% of the current potential range) (Figure S3.2 in Appendix S3). *D. camerooniana* will experience a markedly lower loss rate in the potential distribution area if the optimistic RCP 2.6 scenario occurs (27.6%). However, if the pessimistic/negative RCP 8.5 scenario occurs, the loss rate will reach a similar level as for *D. afromontana* (60.5%) (Table 3). The RCP 2.6 climate change scenario seems to affect the *D. camerooniana* potential range relatively slightly. In contrast, at RCP 8.5, most countries lose large fractions of this range, especially DR Congo, which is predicted to lose at least 60% of the current potential range of *D. camerooniana* (Figure S3.2 in Appendix S3).

In contrast, the potential distribution area of *D. surculosa* is projected to increase until 2050 (Figure 5e–f). Under the RCP 8.5 scenario, the median current potential distribution of this species is predicted to be almost two times larger than at present, with especially high increases in Ivory Coast, Cameroon, and Liberia. In Ghana and Sierra Leone, the *D. surculosa* area increases only if the RCP 8.5 scenario is applied. In addition, the *D. surculosa* area in Nigeria is expected to only slightly increase regardless of the climate change scenario (Table 3, Figure S3.2).

## 4 DISCUSSION AND CONCLUSIONS

### 4.1 Potential distribution modeling in *Dracaena* species

To date, the potential impact of climate change on the *Dracaena* species has been analyzed only for two species from the “dragon tree” group: *Dracaena cinnabari* Balf.f. from Socotra (Yemen), and *Dracaena ombet* Kotschy and Peyr. from northeastern Africa (Attorre et al., 2007; Robiansyah & Hajar, 2017). Robiansyah and Hajar (2017) used a Maxent model to estimate the current and future distribution of *D. ombet* according to the “worst” climate change scenario (RCP 8.5 projections for 2050 and 2070). This species is classified as endangered on the IUCN list, and moreover, the population consists mainly of old specimens, and seedlings are found very rarely. The locations of *D. ombet* are hardly accessible because they occur on steep slopes in the desert mountains of northeastern Africa, so the geographical distribution of the species is poorly known (Elnoby et al., 2017). Furthermore, although *D. ombet* is well adjusted to drought conditions, the future potential extent of this species markedly decreases (Robiansyah & Hajar, 2017). These authors obtained a satisfactory model (AUC = 0.979) despite the study being based on only 24 location records. However, the AUC values for the testing set have not been given, and thus, it is difficult to estimate the real predictive power of this model. In contrast, in our study, we used many more locations (at least 122 records for *D. afromontana*), and our model was of a similar quality at training set (AUC = 0.981 and AUC = 0.966, for test and training set, respectively). A similar methodology, but using regression tree analysis instead of Maxent, has been used to assess the changes in the potential distribution of *D. cinnabari* in Socotra (Attorre et al., 2007). It turned out that this species is also predicted to lose its potential habitat in the future.

The species *D. cinnabari* and *D. ombet* are considered remnants from the Mio‐Pliocene subtropical forests and are considered close to extinction because of the late Pliocene climate changes causing the drying and desertification of northwestern Africa (Mies, 1996). The question is whether the forest understory plants from the *Dracaena* genus growing in tropical Africa will be affected by such dramatic climate changes, which will possibly lead to their extinction. Until now, *Dracaena* forest undergrowth plants were not studied in this way (Pierzchalska et al., 2014). In this study, we chose three endemic African forest undergrowth species characterized by different distributions within the continent but connected with humid habitats. We examined the next 30 years of projected climate changes and showed that selected species respond differently to the climatic signal. *D. afromontana* habitat area will decrease over 50% until 2050 according to both RCP 2.6 and RCP 8.5. We documented that the optimum temperature in the wettest quarter for this species does not exceed 20°C. However, the climatic projections for the region indicate an increase in temperature (Engelbrecht et al., 2015). Thus, the minimum altitude at which *D. afromontana* could grow is increasing. This phenomenon results in a reduction of the area of potential habitats of *D. afromontana*. In contrast, there is evidence that *D. afromontana* may be grown by farmers as a fence or boundary marker (Sheridan, 2008), which may be the reason that the number of locations of this species will not decrease as rapidly as predicted. Nevertheless, we predicted a future loss of or decrease in potential natural *D. afromontana* habitats. Currently, the status of *D. afromontana* on the IUCN Red list is of Least Concern because the population is relatively stable in Eastern Africa (Kelbessa et al., 2013). However, there is evidence that the potential distribution area of *D. afromontana*, the Albertine Rift, is likely to lose many endemic species until 2080 (specifically, up to 80% of the current ranges of endemic plants are projected to be lost) (Ayebare, Plumptre, Kujirakwinja, & Segan, 2018). Therefore, *D. afromontana* should be treated as a species that is the most vulnerable to projected climate warming.

Potential habitats for *D. camerooniana* seem to be less vulnerable to climate warming, but the model developed in this study showed a possible slight decrease until 2050. However, changing the scenario from RCP 2.6 to RCP 8.5 greatly increases the loss in potential range area (from 27% to 60%, respectively). Currently, the species distribution area is relatively large, *D. camerooniana* is frequently found in different types of forests, including secondary and degraded forests, and the population is considered generally stable (Crook, 2013). We suggest that forest loss may exert a stronger impact than climate warming in the near future.

In contrast to the previous species, the range of *D. surculosa* is predicted to increase under both climate conditions analyzed. In particular, an expansion toward Central Africa is expected. We documented that low temperature and precipitation can limit the growth of *D. surculosa*, but the predicted future changes result in the opposite conditions, so we assume that this species is not endangered when considering climatic factors only.

### 4.2 Discrepancies between the number of herbarium records and current potential distribution

In some countries, relatively few locations of *Dracaena* species were recorded. Nevertheless, the record density per 10,000 km^2^ was higher than that in the remaining countries. This was the case with *D. afromontana* in Burundi and Rwanda, *D. camerooniana* in Equatorial Guinea, and *D. surculosa* in Liberia and Sierra Leone. This result may imply that these countries have been well surveyed for *Dracaena* species. The common feature of the mentioned countries is that they are rather small and are located in the center of the known geographic ranges of the particular *Dracaena* species. Thus, large parts of these countries constitute suitable habitats for the species, which is another explanation of the high record density in these countries.

We noted that the current potential distribution range was also modeled in countries in which *Dracaena* species location records were scarce or absent. A small number or no records of *D. camerooniana* originated from Eastern Africa (Ethiopia, Kenia, Uganda, Tanzania, Malawi, and Mozambique) but the Maxent model indicated some isolated, species‐suitable areas in central Ethiopia and southern Tanzania. Similarly, *D. surculosa* herbarium material came from locations gathered around the Gulf of Guinea, with Cameroon and Nigeria as the easternmost countries. However, the *D. surculosa* model revealed that a potential range may also occur further to the east, especially in the Central African Republic and DR Congo. All these examples confirm that the modeled potential range area may overestimate the current *Dracaena* species distribution. Nonetheless, these “unexpected” modeled areas may become new focus territories for future niches and population discoveries. However, one needs to keep in mind that the suitability of an area does not necessarily mean that it is accessible to the species. The current and past distribution of habitat types that are unsuitable for the species in question might create impenetrable biological barriers. Additionally, the dispersal mode might prevent the species from gaining a wider range. *Dracaena* species generally have fleshy fruits and are assumed to be dependent on primates, birds, and rodents for successful seed dispersal (Bollen & Van Elsacker, 2002; González‐Castro et al., 2019; Mwachala, 2005). Since Africa is known to have undergone dramatic climate changes in its recent geological history (Anhuf, 2000), a lack of suitable paths of migration could have prevented the species from reaching areas suitable for their survival.

Surprisingly, *D. camerooniana*, having a well‐defined geographic distribution with a primary center located around the Gulf of Guinea and in the Congo Basin, also has a secondary center of distribution that differs markedly in terms of environmental characteristics from the primary center. The secondary center of the range, the Katanga Plateau, is located at a much higher altitude (approximately 2,000 m a.s.l.) and latitude (15°S) than the Congo Basin. Although one important environmental variable for the potential distribution of *D. camerooniana*, prec_wet, is very similar in the Congo Basin and on the Katanga Plateau, the second crucial variable, temp_range, differs considerably at the Katanga Plateau, being approximately 10°C higher than in the Congo Basin. This suggests that *D. camerooniana* can survive in various climate conditions. Additionally, *D. surculosa* can be found outside its geographical distribution center in the Guineo‐Sudanian bioregion but at higher altitudes than in the Guineo‐Congolian bioregion. Further research is recommended to explore possible genetic and ecological adaptations of *D. camerooniana* on the Katanga Plateau in relation to its major distribution center in the Congo Basin.

## 5 CONCLUDING REMARKS

An understanding, documentation, and recognition of all plant diversity is one of the targets of the Global Strategy for Plant Conservation, which is a program of the Convention on Biological Diversity (http://www.plants2020.net/about-the-gspc/). There is a need to understand the ecology and habitat requirements of species closely related to cultivated plants, also ornamental plants. The importance of such studies is connected with possible resistance to pests and diseases, which is present in wild species but might not be found in the genomes of the cultivated species. Defining the ecogeographic characteristics of such species can lead to the characterization of such potentially important adaptive traits (Heywood, Casas, Ford‐Lloyd, Kell, & Maxted, 2007). In examining the distribution of *Dracaena* species, one needs, however, to remember that the cultural significance of this genus in Africa is immense, and some species, for example, *D. arborea,* are planted on purpose in populated areas to mark boundaries, grave sites, and other important places (Sheridan, 2008). Additionally, *D. afromontana* is known to occur in farmlands in Rwanda, where this species is used as a source of fiber (Smith et al., 2018). The list of the species used for different cultural and traditional purposes is so far incomplete, except for the information which can be gathered from the herbarium sheets.

To conclude, this is the first study to estimate the current and future (year 2050) potential distribution of the selected forest undergrowth *Dracaena* Vand. ex L. species. For *D. afromontana* Mildbr., *D. camerooniana* Baker, and *D. surculosa* Lindl., we showed how the probability of species occurrence changes throughout Africa. The outcome shows that species occupying similar habitats may differently respond to similar climatic changes. While the first two species show a decrease in their ranges, the latter actually gains niches with projected climate changes. Moreover, the great importance of *Dracaena* species in human cultivation and culture might add positively to their distribution in anthropogenic habitats. More studies concerning spatial distribution and its future changes in *Dracaena* species are needed, as this understanding is necessary for the protection of these important iconic species and the richest diversity centers of these useful plants. This understanding is also potentially helpful to assess the climate change vulnerability of the entire group of forest undergrowth plants.

## CONFLICT OF INTEREST

None declared.

## AUTHOR CONTRIBUTIONS

PB and JWS conceived the ideas; TD and JWS validated herbarium materials; TD, PB, KP, MN and JP prepared a database; PB and JWS analyzed the data; and PB and JWS led the writing with assistance from TD. All authors contributed to the interpretation of results and provided comments on the manuscript.

## Data Availability

Climate data from Worldclim database were used (worldclim.org). Sampling locations (Maxent input files) as well as GIS layers showing the locations are stored in Dryad repository (https://doi.org/10.5061/dryad.96240sr).

## References

[ece35251-bib-0001] Ahmed, A. G. M. , & Mlay, W. (1998). Environment and sustainable development in Eastern and Southern Africa: Some critical issues. New York, NY: St. Martin's Press, Inc, Scholarly and Reference Division.

[ece35251-bib-0002] Angiosperm Phylogeny Group (APG) (2009). An update of the Angiosperm Phylogeny Group classification for the orders and families of flowering plants: APG III. Botanical Journal of the Linnean Society, 161, 105–121.

[ece35251-bib-0003] Anhuf, D. (2000). Vegetation history and climate changes in Africa North and South of the Equator (10°S to 10°N) during the Last Glacial Maximum In Smolka P., & Volkheimer W. (Eds.), Southern Hemisphere Paleo‐ and Neoclimates (pp. 225–248). Berlin, Heidelberg: Springer.

[ece35251-bib-0004] Apaydin, H. , Anli, A. S. , & Ozturk, F. (2011). Evaluation of topographical and geographical effects on some climatic parameters in the Central Anatolia Region of Turkey. International Journal of Climatology, 31, 1264–1279. 10.1002/joc.2154

[ece35251-bib-0005] Attorre, F. , Francesconi, F. , Taleb, N. , Scholte, P. , Saed, A. , Alfo, M. , & Bruno, F. (2007). Will dragonblood survive the next period of climate change? Current and future potential distribution of *Dracaena cinnabari* (Socotra, Yemen). Biological Conservation, 138, 430–439. 10.1016/j.biocon.2007.05.009

[ece35251-bib-0006] Ayebare, S. , Plumptre, A. J. , Kujirakwinja, D. , & Segan, D. (2018). Conservation of the endemic species of the Albertine Rift under future climate change. Biological Conservation, 220, 67–75.

[ece35251-bib-0007] Baker, T. R. , Phillips, O. L. , Malhi, Y. , Almeida, S. , Arroyo, L. , Di Fiore, A. , … Vásquez Martínez, R. (2004). Increasing biomass in Amazonian forest plots. Philosophical Transactions of the Royal Society of London. Series B: Biological Sciences, 359, 353–365. 10.1098/rstb.2003.1422 15212090 PMC1693327

[ece35251-bib-0008] Bollen, A. , & Van Elsacker, L. (2002). Feeding ecology of Pteropus rufus (Pteropodidae) in the Littoral Forest of Sainte Luce, SE Madagascar. Acta Chiropterologica, 4(1), 33–47.

[ece35251-bib-0009] Bos, J. J. (1984). *Dracaena* in West Africa. Agricultural University Wageningen Papers 84‐1, The Netherlands, pp. 6833–126.

[ece35251-bib-0010] Bos, J. J. (1998). Dracaenaceae. In Kubitzki K. (Ed.), The Families and genera of flowering plants, monocotyledons, lilianae (Except Orchidacea), pp. 238–241. Berlin, Germany: Springer.

[ece35251-bib-0011] Brands, S. , Herrera, S. , Fernandez, J. , & Gutierrez, J. M. (2013). How well do CMIP5 Earth System Models simulate present climate conditions in Europe and Africa? A performance comparison for the downscaling community. Climate Dynamics, 41, 803–817. 10.1007/s00382-013-1742-8

[ece35251-bib-0012] Christensen, R. (2002). Plane answers to complex questions: The theory of linear models. (Springer Texts in Statistics). New York, NY: Springer.

[ece35251-bib-0013] Clark, D. A. , Clark, D. B. , & Oberbauer, S. F. (2013). Field‐quantified responses of tropical rainforest aboveground productivity to increasing CO2 and climatic stress, 1997–2009. Journal of Geophysical Research: Biogeosciences, 118, 783–794.

[ece35251-bib-0014] Collins, W. J. , Bellouin, N. , Doutriaux‐Boucher, M. , Gedney, N. , Halloran, P. , Hinton, T. , … Woodward, S. (2011). Development and evaluation of an Earth‐System model – HadGEM2. Geoscientific Model Development, 4, 1051–1075. 10.5194/gmd-4-1051-2011

[ece35251-bib-0015] Costion, C. M. , Simpson, L. , Pert, P. L. , Carlsen, M. M. , Kress, W. J. , & Crayn, D. (2015). Will tropical mountaintop plant species survive climate change? Identifying key knowledge gaps using species distribution modelling in Australia. Biological Conservation, 191, 322–330. 10.1016/j.biocon.2015.07.022

[ece35251-bib-0016] Crawford, P. H. C. , & Hoagland, B. W. (2009). Can herbarium records be used to map alien species invasion and native species expansion over the past 100 years? Journal of Biogeography, 36(4): 651–661.

[ece35251-bib-0017] Crook, V. (2013). Dracaena camerooniana. The IUCN Red List of Threatened Species, 2013, e.T44393573A44477731 10.2305/IUCN.UK.2013-2.RLTS.T44393573A44477731.en

[ece35251-bib-0018] Cuyckens, G. A. , Christie, D. A. , Domic, A. I. , Malizia, L. R. , & Renison, D. (2016). Climate change and the distribution and conservation of the world's highest elevation woodlands in the South American Altiplano. Global and Planetary Change, 137, 79–87. 10.1016/j.gloplacha.2015.12.010

[ece35251-bib-0019] Damen, T. H. J. , van der Burg, W. J. , Wiland‐Szymańska, J. , & Sosef, M. S. (2018). Taxonomic novelties in African *Dracaena* (Dracaenaceae). Blumea ‐ Biodiversity, Evolution and Biogeography of Plants, 63(1), 31–53. 10.3767/blumea.2018.63.01.05

[ece35251-bib-0020] Di Febbraro, M. , Carotenuto, F. , Castiglione, S. , Russo, D. , Loy, A. , Maiorano, L. , & Raia, P. (2017). Does the jack of all trades fare best? Survival and niche width in Late Pleistocene megafauna. Journal of Biogeography, 44(12), 2828–2838. 10.1111/jbi.13078

[ece35251-bib-0021] Dike, V. N. , Shimizu, M. H. , Diallo, M. , Lin, Z. , Nwofor, O. K. , & Chineke, T. C. (2015). Modelling present and future African climate using CMIP5 scenarios in HadGEM2‐ES. International Journal of Climatology, 35, 1784–1799. 10.1002/joc.4084

[ece35251-bib-0022] Droissart, V. , Dauby, G. , Hardy, O. J. , Deblauwe, V. , Harris, D. J. , Janssens, S. , … Couvreur, T. L. P. (2018). Beyond trees: Biogeographical regionalization of tropical Africa. Journal of Biogeography, 45(5), 1153–1167. 10.1111/jbi.13190

[ece35251-bib-0023] Elith, J. , Graham, C. H. , & Anderson, R. P. , Dudík, M. , Ferrier, S. , Guisan, A. , … Zimmermann, E. N. (2006). Novel methods improve prediction of species' distributions from occurrence data. Ecography, 29, 129–151. 10.1111/j.2006.0906-7590.04596.x

[ece35251-bib-0024] Elith, J. , & Leathwick, J. (2007). Predicting species distributions from museum and herbarium records using multiresponse models fitted with multivariate adaptive regression splines. Diversity and Distributions, 13(3), 265–275. 10.1111/j.1472-4642.2007.00340.x

[ece35251-bib-0025] Elith, J. , Phillips, S. J. , Hastie, T. , Dudík, M. , Chee, Y. E. , & Yates, C. J. (2011). A statistical explanation of MaxEnt for ecologists. Diversity and Distributions, 17, 43–57. 10.1111/j.1472-4642.2010.00725.x

[ece35251-bib-0026] Elnoby, S. K. , Moustafa, A. A. , & Mansour, S. R. (2017). Impact of climate change on the endangered Nubian dragon tree (*Dracaena ombet*) in the South Eastern of Egypt. Catrina, 16(1), 23–28.

[ece35251-bib-0027] Engelbrecht, F. , Adegoke, J. , Bopape, M.‐J. , Naidoo, M. , Garland, R. , Thatcher, M. , … Gatebe, C. (2015). Projections of rapidly rising surface temperatures over Africa under low mitigation. Environmental Research Letters, 10, 85004 10.1088/1748-9326/10/8/085004

[ece35251-bib-0028] FAO (2001). Forest Resources Assessment 2000. FAO Forestry Papers 140, Rome.

[ece35251-bib-0029] Fauset, S. , Baker, T. R. , Lewis, S. L. , Feldpausch, T. R. , Affum‐Baffoe, K. , Foli, E. G. , … Swaine, M. D. (2012). Drought‐induced shifts in the floristic and functional composition of tropical forests in Ghana. Ecology Letters, 15, 1120–1129. 10.1111/j.1461-0248.2012.01834.x 22812661

[ece35251-bib-0030] Fithian, W. , & Hastie, T. (2013). Finite‐sample equivalence in statistical models for presence‐only data. Annals of Applied Statistics, 7(4), 1917–1939. 10.1214/13-AOAS667 25493106 PMC4258396

[ece35251-bib-0031] Franklin, J. , & Miller, J. A. (2009). Mapping species distributions: Spatial inference and prediction. New York, NY: Cambridge University Press.

[ece35251-bib-0032] González‐Castro, A. , Pérez‐Pérez, D. , Romero, J. , & Nogales, M. (2019). Unraveling the seed dispersal system of an insular “Ghost” Dragon Tree (*Dracaena draco*) in the wild. Frontiers in Ecology and Evolution, 7(39), 6833–11.

[ece35251-bib-0033] Govaerts, R. (2018). World Checklist of Asparagaceae In Roskov Y., Abucay L., Orrell T., … Penev L. (Eds.), Species 2000 & ITIS Catalogue of Life, 28th March 2018. World Checklist of Selected Plant Families (version Aug 2017). Digital resource at www.catalogueoflife.org/col. Species 2000. Leiden, The Netherlands: Naturalis.

[ece35251-bib-0034] Hanley, J. A. , & McNeil, B. J. (1982). The meaning and use of the area under a receiver operating characteristic (ROC) curve. Radiology, 143(1), 29–36. 7063747 10.1148/radiology.143.1.7063747

[ece35251-bib-0035] Heywood, V. , Casas, A. , Ford‐Lloyd, B. , Kell, S. , & Maxted, N. (2007). Conservation and sustainable use of crop wild relatives. Agriculture, Ecosystems and Environment, 121(2007), 245–255. 10.1016/j.agee.2006.12.014

[ece35251-bib-0036] Hijmans, R. J. , Cameron, S. E. , Parra, J. L. , Jones, P. G. , & Jarvis, A. (2005). Very high resolution interpolated climate surfaces for global land areas. International Journal of Climatology, 25(15), 1965–1978. 10.1002/joc.1276

[ece35251-bib-0037] Jaines, E. T. (1957). Information theory and statistical mechanics. Physics Reviews, 106, 620–630.

[ece35251-bib-0038] James, R. , Washington, R. , & Rowell, D. P. (2013). Implications of global warming for the climate of African rainforests. Philosophical Transactions of the Royal Society B: Biological Sciences, 368, 20120298. 10.1098/rstb.2012.0298 PMC3720020 23878329

[ece35251-bib-0039] Jura‐Morawiec, J. , & Tulik, M. (2016). Dragon's blood secretion and its ecological significance. Chemoecology, 26, 101–105. 27239099 10.1007/s00049-016-0212-2 PMC4863904

[ece35251-bib-0040] Kelbessa, E. , Kalema, J. , & Crook, V. (2013). Dracaena afromontana. The IUCN Red List of Threatened Species, 2013, e.T44392757A44413063 10.2305/IUCN.UK.2013-2.RLTS.T44392757A44413063.en

[ece35251-bib-0041] Kelly, A. E. , & Goulden, M. L. (2008). Rapid shifts in plant distribution with recent climate change. Proceedings of the National Academy of Sciences of the United States of America, 105(33), 11823–11826. 18697941 10.1073/pnas.0802891105 PMC2575286

[ece35251-bib-0042] Kukwa, M. , & Kolanowska, M. (2016). Glacial refugia and the prediction of future habitat coverage of the South American lichen species *Ochrolechia austroamericana* . Scientific Reports, 6(1), 38779 10.1038/srep38779 27929090 PMC5144090

[ece35251-bib-0043] Laurance, W. F. , & Cochrane, M. A. (2001). Special section: Synergistic effects in fragmented landscapes. Conservation Biology, 15, 1488–1489.

[ece35251-bib-0044] Lewis, S. L. (2006). Tropical forests and the changing earth system. Philosophical Transactions of the Royal Society B: Biological Sciences, 361, 195–210. 10.1098/rstb.2005.1711 PMC1626535 16553317

[ece35251-bib-0045] Lewis, S. L. , Malhi, Y. , & Phillips, O. L. (2004). Fingerprinting the impacts of global change on tropical forests. Philosophical Transactions of the Royal Society of London. Series B: Biological Sciences, 359, 437–462. 10.1098/rstb.2003.1432 15212095 PMC1693339

[ece35251-bib-0046] Liu, C. , White, M. , & Newell, G. (2013). Selecting thresholds for the prediction of species occurrence with presence‐only data. Journal of Biogeography, 40, 778–789. 10.1111/jbi.12058

[ece35251-bib-0047] Lloyd, J. , & Farquhar, G. D. (2008). Effects of rising temperatures and [CO2] on the physiology of tropical forest trees. Philosophical Transactions of the Royal Society of London. Series B: Biological Sciences, 363, 1811–1817. 18267901 10.1098/rstb.2007.0032 PMC2374913

[ece35251-bib-0048] Lu, P.‐L. , & Morden, C. (2010). Phylogenetics of plant genera *Dracaena* and *Pleomele* (Asparagaceae). Botanica orientalis – Journal of Plant Science, 7, 64–72.

[ece35251-bib-0049] Marrero, A. , Almeida, R. S. , & González‐Martín, M. (1998). A new species of the wild dragon tree, Dracaena (Dracaenaceae) from Gran Canaria and its taxonomic and biogeographic implications. Botanical Journal of the Linnean Society, 128, 291–314.

[ece35251-bib-0050] Mies, B. A. (1996). The phytogeography of Socotra: evidence for disjunctive taxa, especially with Macaronesia In Dumont H. J. (Ed.), Proceedings of the First International Symposium on Socotra Island: Present and future (pp. 83–105). New York, NY: United Nations Publications.

[ece35251-bib-0051] Moss, R. H. , Edmonds, J. A. , Hibbard, K. A. , Manning, M. R. , Rose, S. K. , van Vuuren, D. P. , … Wilbanks, T. J. (2010). The next generation of scenarios for climate change research and assessment. Nature, 463, 747–756. 10.1038/nature08823 20148028

[ece35251-bib-0052] Mwachala, G. (2005). Systematics and ecology of *Dracaena* L. (Ruscaceae) in Central, East and Southern Africa. PhD thesis, manuscript. Institute of Biology, University of Koblenz‐Landau.

[ece35251-bib-0053] Newbold, T. (2010). Applications and limitations of museum data for conservation and ecology, with particular attention to species distribution models. Progress in Physical Geography: Earth and Environment, 34, 3–22. 10.1177/0309133309355630

[ece35251-bib-0054] Okunji, C. O. , Iwu, M. M. , Jackson, J. E. , & Tally, J. D. (1996). Biological activity of saponins from two *Dracaena* species. Advances in Experimental Medicine and Biology, 404, 415–428. 8957311 10.1007/978-1-4899-1367-8_33

[ece35251-bib-0055] Pearson, G. R. , & Dawson, T. P. (2003). Predicting the impacts of climate change on the distribution of species: Are bioclimate envelope models useful? Global Ecology & Biogeography, 12, 361–371. 10.1046/j.1466-822X.2003.00042.x

[ece35251-bib-0056] Phillips, S. J. , Anderson, R. P. , & Schapire, R. E. (2006). Maximum entropy modeling of species geographic distributions. Ecological Modelling, 190, 231–259.

[ece35251-bib-0057] Phillips, S. J. , & Dudik, M. (2008). Modeling of species distributions with Maxent: New extensions and a comprehensive evaluation. Ecography, 31, 161–175. 10.1111/j.0906-7590.2008.5203.x

[ece35251-bib-0058] Pierzchalska, J. , Nowak, M. M. , Wilkin, P. , Mwachala, G. , & Wiland‐Szymańska, J. (2014). Geoenvironmental modelling of the geographic range structure of *Dracaena aubryana* Brongn. Ex C.J. Morren (Asparagaceae) on the African Continent. Badania Fizjograficzne R.V. Seria B Botanika, B63, 7–20.

[ece35251-bib-0059] Ponce‐Reyes, R. , Plumptre, A. J. , Segan, D. , Ayebare, S. , Fuller, R. A. , Possingham, H. P. , & Watson, J. E. M. (2017). Forecasting ecosystem responses to climate change across Africa's Albertine Rift. Biological Conservation, 209, 464–472. 10.1016/j.biocon.2017.03.015

[ece35251-bib-0060] Robiansyah, I. , & Hajar, A. S. (2017). Predicting current and future distribution of endangered tree *Dracaena ombet* Kotschy and Peyr. under climate change. Proceedings of the National Academy of Sciences, India Section B: Biological Sciences, 87(1), 225–232.

[ece35251-bib-0061] Sheridan, M. (2008). Tanzanian ritual perimetrics and African landscapes: The case of *Dracaena* . International Journal of African Historical Studies, 41(3), 491–521.

[ece35251-bib-0062] Shi, H. , Paull, D. , & Rayburg, S. (2016). Spatial heterogeneity of temperature across alpine boulder fields in New South Wales, Australia: Multilevel modelling of drivers of microhabitat climate. International Journal of Biometeorology, 60(7), 965–976. 10.1007/s00484-015-1089-4 26511483

[ece35251-bib-0063] Singh, H. P. , & Dadlani, N. K. (2000). Current status of floriculture – National and International Scenario. New Delhi, India: Commercial Floriculture, Malhotra Publishing House.

[ece35251-bib-0064] Smith, D. E. , Gassner, A. , Agaba, G. , Nansamba, R. , & Sinclair, F. (2018). The utility of farmer ranking of tree attributes for selecting companion trees in coffee production systems. Agroforestry Systems, 6833–15. 10.1007/s10457-018-0257-z

[ece35251-bib-0065] Staples, G. W. , & Herbst, D. R. (2005). A tropical garden Flora. Honolulu, Hawaii: Bishop Museum Press.

[ece35251-bib-0066] Swets, J. A. (1988). Measuring the accuracy of diagnostic systems. Science, 240(4857), 1285–1293. 10.1126/science.3287615 3287615

[ece35251-bib-0067] Syfert, M. M. , Smith, M. J. , & Coomes, D. A. (2013). The effects of sampling bias and model complexity on the predictive performance of MaxEnt species distribution models. PLoS ONE, 8(2), e55158 10.1371/journal.pone.0055158 23457462 PMC3573023

[ece35251-bib-0068] Taylor, K. E. , Stouffer, R. J. , & Meehl, G. A. (2012). An overview of CMIP5 and the experiment design. Bulletin of American Meteorological Society, 93(4), 485–498. 10.1175/BAMS-D-11-00094.1

[ece35251-bib-0069] Ter Steege, H. , Pitman, N. , Sabatier, D. , Castellanos, H. , Van derHout, P. , Daly, D. C. , … Morawetz, W. (2003). A spatial model of tree alphadiversity and tree density for the Amazon. Biodiversity and Conservation, 12, 2255–2277.

[ece35251-bib-0070] Thiers, B. (2018). Index Herbariorum: A global directory of public herbaria and associated staff. New York Botanical Garden's Virtual Herbarium. Retrieved from http://sweetgum.nybg.org/science/ih/

[ece35251-bib-0071] Vedel‐Sørensen, M. , Tovaranonte, J. , Bøcher, P. K. , Balslev, H. , & Barfod, A. S. (2013). Spatial distribution and environmental preferences of 10 economically important forest palms in western South America. Forest Ecology and Management, 307, 284–292. 10.1016/j.foreco.2013.07.005

[ece35251-bib-0072] Warren, D. L. , Glor, R. E. , & Turelli, M. (2010). ENMTools: A toolbox for comparative studies of environmental niche models. Ecography, 33(3), 607–611. 10.1111/j.1600-0587.2009.06142.x

[ece35251-bib-0073] Wiens, J. A. , Stralberg, D. , Jongsomjit, D. , Howell, C. A. , & Snyder, M. A. (2009). Niches, models, and climate change: Assessing the assumptions and uncertainties. Proceedings of the National Academy of Sciences of the United States of America, 106(Suppl. 2), 19729–19736. 10.1073/pnas.0901639106 19822750 PMC2780938

[ece35251-bib-0074] Zhou, L. , Tian, Y. , Myneni, R. B. , Ciais, P. , Saatchi, S. , Liu, Y. Y. , … Hwang, T. (2014). Widespread decline of Congo rainforest greenness in the past decade. Nature, 509, 86–90. 24759324 10.1038/nature13265

