# Supplementary material for: Current and future potential distributions of three Dracaena Vand. ex L. species under two contrasting climate change scenarios in Africa
Source: Ecol Evol. 2019 Jun 11;9(12):6833–48. doi: 10.1002/ece3.5251 (PMC6662280; doi:10.1002/ece3.5251)
Supplement: Supplementary file 1 [file ECE3-9-6833-s001.docx]

Appendix 1

Table S1.1. Herbaria of the origin of occurrence records considered in the study

| Abbreviation | Institution | Location |
| --- | --- | --- |
| A | Harvard University | Massachusetts, Cambridge, the USA |
| AAU | Aarhus University | Aarhus, Denmark |
| ABI | Centre ORSTOM d'Adiopodoume | Abidjan, Ivory Coast |
| ACD | Alemaya University of Agriculture | Dire Dawa, Ethiopia |
| AMD | National Herbarium of the Netherlands, Hugo de Vries-Laboratory | Leiden, the Netherlands |
| B | Botanischer Garten und Botanisches Museum Berlin-Dahlem, Zentraleinrichtung der Freien Universität Berlin | Berlin, Germany |
| BENIN | Université National du Bénin | Cotonou, Benin |
| BISH | Bishop Museum | Honolulu, Hawaii, the USA |
| BM | The Natural History Museum | London, England, UK |
| BO | Research Centre for Biology | Cibinong, Indonesia |
| BP | Hungarian Natural History Museum | Budapest, Hungary |
| BR | Botanic Garden Meise | Meise, Belgium |
| BRLU | Université Libre de Bruxelles | Bruxelles, Belgium |
| C | University of Copenhagen | Copenhagen, Denmark |
| CNS | Australian Tropical Herbarium | Cairns, Queensland, Australia |
| COI | University of Coimbra | Coimbra, Portugal |
| CONN | University of Connecticut | Storrs, Connecticut, the USA |
| CSRS | Centre Suisse de Recherches Scientifiques en Côte d’Ivoire | Abidjan, Ivory Coast |
| DES | Desert Botanical Garden | Phoenix, Arizona, the USA |
| E | Royal Botanic Garden Edinburgh | Edinburgh, Scotland, UK |
| EA | National Museums of Kenya | Nairobi, Kenya |
| ENT | Ministry of Natural Resources | Entebbe, Uganda |
| ETH | Addis Ababa University | Addis Ababa, Ethiopia |
| FH | Harvard University | Massachusetts, Cambridge, the USA |
| FHI | Forestry Research Institute of Nigeria | Ibadan, Nigeria |
| FHO | University of Oxford | Oxford, England, UK |
| FI | Natural History Museum | Firenze, Italy |
| FLAS | Florida Museum of Natural History | Gainesville, Florida, the USA |
| FMB | Instituto de Investigación de Recursos Biológicos Alexander von Humboldt | Santafé de Bogotá, Colombia D.C. |
| FR | Senckenberg Gesellschaft für Naturforschung: Senckenberg Forschungsinstitut und Naturmuseum | Frankfurt, Germany |
| FT | Centro Studi Erbario Tropicale, Università degli Studi di Firenze | Firenze, Italy |
| G | Conservatoire et Jardin botaniques de la Ville de Genève | Genève, Switzerland |
| GC | University of Ghana | Legon, Ghana |
| GENT | Ghent University | Ghent, Belgium |
| GH | Harvard University | Massachusetts, Cambridge, the USA |
| HBG | University of Hamburg | Hamburg, Germany |
| HNG | Université. Gamal Abdel Nasser de Conakry | Conakry, Republic of Guinea |
| HUJ | Hebrew University | Jerusalem, Israel |
| IAGB | Botanical Garden "Anastasie Fatu" of Iasi | Iasi, Romania |
| IEC | Centre D'Etude sur les Ressources Végétales | Brazzaville, Congo Republic |
| IFAN | Institut Fondamental d'Afrique Noire | Dakar, Senegal |
| IFE | Obafemi Awolowo University | Ile-ife, Nigeria |
| IRSC | Institut de Recherches Scientifiques au Congo | Brazzaville, Congo Republic |
| K | Royal Botanic Gardens | Kew, England, UK |
| KHF | Forest Research and Education Institute, Soba | Khartoum, Sudan |
| KIS | Université de Kisangani | Kisangani, Democratic Republic of the Congo |
| KRIB | Korea Research Institute of Bioscience and Biotechnology | Daejeon, Republic of Korea |
| L | Naturalis | Leiden, the Netherlands |
| LBV | CENAREST | Libreville, Gabon |
| LD | Lund University | Lund, Sweden |
| LG | Université de Liège | Liège, Belgium |
| LIB | University of Liberia | Monrovia, Liberia |
| LISC | Instituto de Investigação Científica Tropical | Lisboa, Portugal |
| LISU | Museu Nacional de História Natural e da Ciência | Lisboa, Portugal |
| LMU | Eduardo Mondlane University | Maputo, Mozambique |
| M | Botanische Staatssammlung München | München, Germany |
| MA | Real Jardín Botánico | Madrid, Spain |
| MBM | Museu Botânico Municipal | Curitiba, Brazil |
| MO | Missouri Botanical Garden | Saint Louis, Missouri, the USA |
| MOG | National Range Agency | Mogadishu, Somalia |
| MPU | Université de Montpellier | Montpellier, France |
| MU | Miami University | Oxford, Ohio, the USA |
| MHU | Makerere University | Kampala, Uganda |
| NAI | University of Nairobi | Nairobi, Kenya |
| NBG | South African National Biodiversity Institute | Cape Town, South Africa |
| NCSC | North Carolina State University | Raleigh, North Carolina, the USA |
| NDO | Division of Forest Research | Kitwe, Zambia |
| NHT | Tropical Pesticides Research Institute | Arusha, Tanzania |
| NY | The New York Botanical Garden | Bronx, New York, the USA |
| O | Botanical Museum, University of Oslo | Oslo, Norway |
| P | Muséum National d'Histoire Naturelle | Paris, France |
| PAT | Muséum National d'Histoire Naturelle | Paris, France |
| PE | Institute of Botany, Chinese Academy of Sciences | Beijing, People's Republic of China |
| POZG | Adam Mickiewicz University | Poznan, Poland |
| PRE | South African National Biodiversity Institute | Pretoria, South Africa |
| RB | Jardim Botânico do Rio de Janeiro | Rio de Janeiro, Brazil |
| S | Swedish Museum of Natural History | Stockholm, Sweden |
| SCA | Limbe Botanical & Zoological Gardens | Limbe, Cameroon |
| SERG | Institut de Recherche Agronomique de Guinée | Sérédou, Republic of Guinea |
| SL | University of Sierra Leone, Njala University College | Freetown, Sierra Leone |
| SRGH | Botanic Garden | Harare, Zimbabwe |
| U | Naturalis | Leiden, the Netherlands |
| UBT | University of Bayreuth | Bayreuth, Germany |
| UC | University of California | Berkeley, California, the USA |
| UCI | University of Ibadan | Ibadan, Nigeria |
| UCJ | Université d'Abidjan | Abidjan, Ivory Coast |
| UMO | University of Missouri | Columbia, Missouri, the USA |
| UPS | Museum of Evolution | Uppsala, Sweden |
| US | Smithsonian Institution | D.C.Washington, the USA |
| W | Naturhistorisches Museum Wien | Wien, Austria |
| WAG | Naturalis | Leiden, the Netherlands |
| YA | National Herbarium of Cameroon | Yaoundé, Cameroon |
| Z | Universität Zürich | Zürich, Switzerland |

Appendix 2

Table S2.1. List of validated herbarium records of *Dracaena afromontana* by country of origin. Structure of the table: Collector(s) name, collector number, owning herbarium(a)

**Burundi.** *Auquier 4178* (BR, GENT); *Baudet 296* (BR, K); *Becquet 893* (BR); *Declercq 85* (BR); *Lewalle 1231* (BR, EA, MO), *1272* (BR, K, MO), *1511* (BR, K, MO) & *4056* (BR, G, K, MO); *Reekmans 882* (BR, EA, MO)*, 2374* (BR, EA, MO, SRGH) & *10102* (BR, K, MO, WAG); *Robyns 2362* (BR, NY, P, WAG); *Symoens 935* (BRLU, WAG).

**Democratic Republic of Congo.** *Auquier 2174* (BR, GENT); *Bamps 155* (BR, IFAN, WAG); *Bequaert 3777* (BR) & *4293* (BR); *Bokdam 3550* (WAG); *Burtt 3131* (EA, K); *Froment 683* (BR, K); *Germain 1368* (BR) & *3478* (BR, K); *Hauman 11* (BRLU, WAG); *Hendrickx 3216* (BR, EA, PRE) & *3700* (BR, EA, PRE); *Humbert 7622* (B, BR, K, P, P, S, WAG); *Léonard 3501* (BR, WAG); *Lebrun 4490* (BR), *4752* (BR, NY, P, WAG) & *4957* (BR); *Lejoly 2560* (BR, BRLU); *Mildbraed 1360* (B, BR, K); *Pierlot 2479* (BR, WAG) & *2720* (BR, WAG); *Spitaels 311* (BR); *Taton 834* (BR); *de Witté & Fredericq 7905* (BR, P) &

9073 (BR).

**Ethiopia**. *Ash 1443* (K); *Bos s.n.* (WAG), *8430* (BR, ETH, MO, WAG), *9378* ( ACD, B, BR, C, E, EA, ETH, FT, G, MO, MPU, O, UPS, WAG), *9436* (WAG) & *9900* (BR, C, ETH, MO, O, WAG); *Burger 2009* (K), *2501* (K) & *2930* (K); *Chaffey 234* (K); *Friis, Hunde & Jacobsen 210* (C, K, WAG); *Friis, Sebsebe Demissew, Assefa Hailu & Ermias Getachew 13286* (C, ETH, K, WAG); *Friis, Aweke, Rasmussen & Vollesen 1832* (C, ETH, K); *Fujimoto 442* (K); *Gillett 5146* (K); *Jansen 2163* (ACD, WAG), *5344* (ACD, ETH, WAG) & *5463* (ACD, ETH, WAG); *Meyer 8056* (FI, K, MO, P, W); *Mooney 6241* (K), *6675* (K) & *8645* (K); *Perdue 6407* (K); *S.c. s.n.* (P); *Sebsebe Demissew, Ensermu Kelbessa & Fikre 4380* (WAG); *Seegeler 2658* (ACD, WAG); *Thulin, Hunde & Tadesse 3643* (K); *Westphal & Westphal-Stevels 3319* (ETH, K, MO, O, WAG); *de Wilde J.J.F.E. 4400* (ACD, B, BR, LMU, MO, MOG, PRE, WAG), *5297* (WAG) & *6291* (ACD, BR, M, MO, WAG); *de Wilde W.J.J.O. & de Wilde-Duyfjes 6780* (WAG), *7695* (WAG), *9325* (BR, K, MO, PRE, WAG), *9923* (BR, K, WAG) & *10245* (WAG).

**Kenya.** *Bally 5615* (K); *Battiscombe 5* (K); *Beecher s.n.* (C, MO, WAG); *Beentje 2020* (EA, WAG); *Brasnett 1356* (K); *Burney & Mathenge 46* (NY, WAG); *Bytebier, Butynski, Ehart, Perkins & Kimaro 21* (BR, EA, K, NAI, WAG); *Dyson 373* (K), *Fries RE & Fries TCE 787* (UPS, WAG) & *1501* (EA, MO, UPS, WAG); *Graham & Bally 5021* (K); *Kamau Wakanene & Mwangangi 66* (MU, NY, WAG); *Maas Geesteranus 5362* (BR, COI, G, K, L, MO, PRE, S, WAG) & *5649* (BR, COI, G, K, L, MO, S, Z); *Napier 2718* (K); *Perdue & Kibuwa 8361* (EA, K, MO, WAG); *Plaizier 1379* (WAG); *Taita Hills Expedition 915* (EA, K, WAG) & *1130* (EA, WAG); *Tweedie 1587* (K); *van Someren 6016* (K); *Vuyk 399* (WAG); *Zogg, Kramer & Gassner 258* (U, Z).

**Malawi.** *Brass 17272* (NY, WAG); *Chapman 1909* (K); *Congdon 335* (K) & *664* (K); *McClounie 183* (K).

**Rwanda.** *Gilbert 2373* (BR, WAG); *Runyinya 794* (BR); *Scaëtta 1721* (A, BR, G, K); *Troupin 15214* (BR) & *16028* (BR, K, WAG).

**South Sudan.** *Myers 10901* (K); *Thomas 1890* (ENT, K); *Vollesen & Friis 396* (C, K, KHF)

**Tanzania.** *Abeid & Kimwaga 2816* (MO); *Drummond & Hemsley 1636* (B, BR, EA, K, LISC, S); *Gereau, Kindeketa & et al. 6373* (MO);

*Gereau, Schmidt & Munyenyembe 5222* (MO, WAG); *Gereau & Greenway & Kabuie 12121 Kayombo 3508* (MO, WAG) & *3932* (MO, WAG); *Goldblatt & Lovett 8236* (K); (BR, EA, K, PRE); *Haarer 1169* (K); *Hemp 604* (UBT), *961* (B, UBT) & *2211* (B, UBT); *Jefford, Juniper & Newbould 1794* (B, BR, K); ); *Jefford, Juniper & Newbould & Mgaza 1737* (B, BR, K); *Kerfoot 333* (K), *3127* (K) & *3344* (BR, EA, PRE); *Kibure 1479* (MO); *Kibure, Abeid & Kawelela 1438* (BR, MO, WAG); *Kindeketa & Mlangwa 854* (MO, POZG, WAG);

*van der Laan & Wubben 1186* (WAG); *Luke W.R.Q, Luke P.A., Mwangoka, Festo & Bodine 11159* (NHT); *Mahinda 139* (K); *Mwangoka 1140* (MO); *Napper 1045* (K); *Njau, Leliyo, Elia, Kayombo, Mboya, Makweta, Olesurutia & al. 369* (NHT) & *402* (NHT); *Peter 680* (B), *1380* (B), *3084* (B, WAG), *16474* (B, WAG), *41822* (B, WAG); *Richards 12196* (BR, K, SRGH); *Ruffo & Kisena 2871* (K) ; *Sacleux 1355* (P); *Schlage 240* (B); *Schlieben 3482* (B, BM, BR, G, LISC, M, PRE, S, Z) & *4565* (B, BM, BR, G, HBG, LISC, M, S, Z); *Stolz 2428* (A, BM, BR, C, EA, FHO, K, K, P, PRE, UPS, Z); *Volkens 1938* (B, BR, K, WAG); *Wiland & Mboya 61* (MO, POZG) & *163* (MO, POZG).

**Uganda.** *Dummer 3604* (K); *Eggeling 3987* (BR, K); *Kerfoot 715* (K); *Lisowski 24326* (BR, POZG, WAG); *Lye 1225* (MHU, O); *Perdue & Kibuwa 8441* (EA, K, MO, WAG); *Snowden 804* (EA, K, MO, P) & *1591* (K); *Thomas 1441* (BR, K), *2361* (BR, K, P) & *2627* (K).

Table S2.2. List of validated herbarium records of *Dracaena camerooniana* by country of origin. Structure of the table: Collector(s) name, collector number, owning herbarium(a)

**Angola.** *Gossweiler 6179* (COI,LISU), *8217* COI, K), *11194* (COI) &*14127* (BM, K); *Welwitsch 3748* (B, BM, COI, G, K, LISU, MO, P, WAG).

**Burundi.** *Auquier 4251* (BR)

**Cameroon.** *Akogo 249* (K, SCA, YA); *Annet 347* (P, WAG) & *411* (P); *Asonganyi 108* (P, WAG, YA) & *298* (P); *Bamps 1374* (BR, WAG) & *1744* (BR); *Bates s.n.* (K) & *s.n.* (K); *Binuyo & Daramola 35552* (FHI, K, WAG); *Bos s.n.* (WAG), *s.n.* (WAG), *3452* (WAG), *3547* (WAG), *3660* (WAG), *3697* (WAG), *3893* (WAG), *3895* (WAG), *4229* (WAG, YA), *4327* (MO, P, WAG, YA), *4340* (WAG), *4682* (WAG, YA), *4683* (WAG), *4684* (WAG), *4685* (WAG), *4686* (WAG), *4687* (WAG), *4688* (WAG, YA), *4689* (MO, WAG, YA), *4690* (BR, MO, WAG, YA), *4691* (WAG), *4692* (MO, WAG, YA), *4693* (WAG), *4694* (WAG), *4695* (WAG), *4696* (WAG), *4697* (WAG), *4698* (BR, C, K, MA, MO, P, PRE, WAG, YA), *4801* (WAG, YA), *4825* (WAG), *4858* (WAG), *4905* (WAG), *4975* (WAG, YA), *5035* (WAG, YA), *5303* (WAG), *5376* (WAG, YA), *5756* (WAG), *6086* (WAG), *6181* (WAG), *6405* (WAG), *6612* (WAG), *6613* (WAG), *6980* (WAG), *7002* (WAG), *7034* (WAG), *7085* (L, WAG, YA), *7407* (WAG) & *10420* (WAG); *Breteler 922* (WAG), *1981* (K, P, WAG, YA), *2673* (P, WAG) & *2974* (P, WAG, YA); *Breyne 5023* (BR); *Cable, Etuge, Faucher M., Faucher P. & Lighava 294* (K,SCA,YA); *Cable, Furneaux, Mukete, & Golembiewski 425* (K, SCA, YA); *Cable, Gawshaw, Solomon, & Jones 550* (K, SCA, WAG, YA); *Cheek, Ndam, Samfon et al. 3232* (K); *Cheek, Scott, Lyonga, Hill, Asser & Williams 5443* (K, SCA, WAG, YA); *Chevalier & Fleury 33415* (P); *Darbyshire, Njume, Ndi, Piggott, Sabirova & Brinklow 313* (K, WAG, YA); *de Kruif 883* (WAG, YA); *de Wilde J.J.F.E. 7593* (BR, MA, MO, P, PRE, WAG, YA), *7891* (BR, EA, K, MA, MO, P, PRE, WAG, YA), *8123* (BR, MO, P, PRE, WAG, YA) & *8256* (WAG); *de Wilde W.J.J.O. & de Wilde-Duyfjes 1851* (P, WAG), *1999* (BR, P, WAG, YA), *2657* (B, BR, K, MO, P, PRE, WAG, YA), *2718* (WAG) & *2781* (BR, K, MO, P, PRE, WAG, YA); *Droissart, Deblauwe, Libalah, Texier, Kamdem, Zébazé & Mofack 1886* (G, BRLU, WAG); *Ejiofor 14032* (K); *Elad, Tchouto Mbatchou, Ekwadi & Nnangah 1499* (KRIBI, SCA, WAG); *Etuge, Greene, Bury & Essama 5170* (K, MO, SCA, WAG, YA); *Jaff 250* (K, SCA, YA); *Khayota 485* (K, SCA, WAG); *Koufani 186* (P, WAG, YA); *Kwangue 135* (K, SCA, YA); *Leeuwenberg* *5995* (AAU, B, BR, EA, FHI, G, K, MA, MBM, MO, P, PRE, SRGH, WAG, YA), *6322* (BR, K, MO, P, PRE, WAG, YA), *8885* (BR, MO, P, WAG), *9125* (MO, WAG), *9151* (WAG), *10611* (BR, MO, P, PRE, UPS, WAG, YA) & *11576* (WAG); *Leeuwenberg & Breteler 8750* (BR, MO, WAG); *Letouzey 1538* (P), *2649* (P), *2835* (P), *4208* (P), *4934* (P), *7445* (BR, COI, K, P, WAG), *8443* (P), *9376* (P, YA), *9546* (P), *9621* (P), *9992* (BR, COI, P)*11274* (BR, K, P, WAG); *11703* (P, WAG, YA); *11921* (P, WAG), *12324* (P, WAG, YA), *13531* (BR, K, P, WAG, YA), *13844* (P), *14337* (P, WAG, YA), *14603* (P) & *15073* (P, WAG, YA); *Letouzey & Villiers* *10423* (WAG) & *10573* (BR, K, P); *Mambo & Thomas 162* (MO, WAG) & *168* (MO, WAG); *Mann 1204* (A, GH, K, P, S, U, WAG); *Mbani 148* (K, SCA, YA); *Mpom 505* (P); *Ndam 653* (K, SCA, YA); *Nemba & Mambo 625* (MO, NY, WAG); *Ngansop, van der Burgt, Álvarez, Hopkins & Tchiengué 235* (BK, WAG, YA); *Nkeng 35* (K, SCA, YA) & *75* (K, SCA, YA); *Nkongmeneck 508* (P, WAG, YA); *Nning, Menyoli & Lyonga 159* (K, SCA, YA); *Onana 13* (WAG, YA); *Plot Series Mt. Cameroon 493* (K, WAG) & *817* (K); *Preuss 146* (K); *Raynal & Raynal-Roques 13473* (P); *Shu Neba 3699* (KRIBI, WAG), *3754* (KRIBI, WAG), *3809* (KRIBI, WAG), *3930* (KRIBI, WAG), *5505* (KRIBI, WAG), *6245* (KRIBI, WAG), *8129* (KRIBI, WAG) & *8622* (KRIBI, WAG); *Shu Neba & Ndoum 1672* (KRIBI, WAG) & *2204* (KRIBI, WAG); *Sidwell, Ebule & Nguembock 107* (K, SCA, YA); *Smits & Mva 112* (KRIBI, WAG), *448* (KRIBI, WAG) & *513* (WAG); *Sonké 1420* (BRLU, WAG); *Sunderland 1028* (K, SCA, YA) & *1333* (K, SCA, YA); *Tchouto Mbatchou 477* (K, SCA, WAG, YA), *539* (K, SCA, YA) & *864* (K, SCA, YA); *Tchouto Mbatchou, Elad, Nganwui & Nkpwele 3069* (KRIBI, SCA, WAG, YA); *Tchouto Mbatchou, Elad & Nnangah 87* (WAG), *107* (WAG), *111* (WAG) & *288* (WAG); *Tekwe 131* (K, SCA, YA); *Tessmann 55* (B, WAG); *van Andel, Kenfack, Maas, Maas-van de Kamer & Jansen-Jacobs 3730* (SCA, WAG, YA); *Thomas 417* (K), *2265* (K, MO, P, WAG), *4872* (MO, P, WAG) & *9893* (K, SCA, YA); *Thomas, Namata, Satabié & Nkongmeneck 7834* (MO, P, WAG); *van Andel, Maas, Maas-van de Kamer, Mva, Jansen-Jacobs & Atou'ou 3644* (KRIBI, WAG, YA); *van der Burgt, Ekwoge, Ngansop, Tchiengué & Xanthos 1662* (K, WAG, YA); *van der Burgt, Tchiengué, Khumbah & Nembou Nong 887* (K, YA); *van Gemerden, Shu Neba & Ndoum 650* (KRIBI, WAG); *Villiers 2369* (WAG,YA) & *2488* (P, WAG); *von Rege 108* (K, SCA, YA); *Watts 180* (K, SCA, YA), *271* (K, SCA, YA), *609* (K, SCA, YA), *718* (K, SCA, YA), *794* (K, SCA, YA) & *817* (K, SCA, YA);*Wheatley 316* (K, SCA, YA), *530* (K, YA) & *734* (K, YA); *Wieringa 1964* (WAG); *Winkler 71* (Z); *Zenker 219* (A, B, BR, C, G, LD, MO, NY, P, U, US, WAG), *2309* (BM, E, G, K, P, WU, Z) & *3809* (BR, GOET, K, L, M, US, WU).

**Central African Republic.** *Allouette s.n.* (L); *Breyne 1524* (BR, LG); *Chevalier 5291* (P), *10925* (P) & *10927* (K, P); *Descoings 12635* (P); *Harris 2261* (MO, WAG), *4085* (BR, MO); *Harris & Fay 1131* (BR, MO, WAG); *Le Testu 3466* (BR, P); *Peeters 166* (PAT); *Tisserant 646* (BM, BR, P), *1922* (P) & *2247* (BR, P, WAG); *Yongo 878* (BRLU, WAG) & *920* (BRLU, WAG); *Zawa 781* (BRLU, WAG).

**Democratic Republic of Congo.** *Achten 310* (BR); *Bavicchi 489* (BR); *Bequaert 2252* (BR, K) & *7459* (BR, K); *Bodenghien 395* (BR); *Bokdam 3666* (KIS, WAG), *4355* (KIS, WAG) & *4537* (WAG); *Bokdam & de Wit 3297* (KIS, WAG); *Bolema 835* (BR); *Breyne 558* (BR), *2558* (BR) & *5210* (BR); *Callens 4161* (BR, NBG); *Christiaensen 2072* (BR); *Compére 23* (BR, WAG) & *1736* (BR); *Cornet d'Elzius, Lauwers & Offerman 666* (BR); *de Bergeyck 1* (BR); *de Giorgi 1069* (BR, WAG); *Delvaux 860* (BR, WAG); *Demeuse 92* (BR); *de Saeger 1423* (BR, WAG) & *1532* (BR, K, WAG); *Desenfans 3820* (BRLU, WAG); *Devred 356* (BR, K, NBG), *746* (BR, WAG) & *1004* (BR, WAG); *Dewévre 701* (BR, BR, WAG); *de Wildeman s.n.* (BR); *Dewulf 190* (BR) & *551* (BR); *de Witte 2571* (BR, EA, LISC, MO, SRGH, WAG), *3755* (BR, WAG) & *6147* (BR, EA, K, WAG); *Dhetchuvi Matchu-Mandje 388* (BRLU, WAG); *Donis 3* (BR), *1851* (BR), *2784* (BR, WAG) & *3288* (BR, WAG); *Dubois 439* (BR) & *440* (BR, EA, K); *Evrard 1237* (BR), *1421* (BR), *2008* (BR), *2138* (BR, EA, WAG), *2766* (BR), *2896* (BR), *2998* (BR, K), *3020* (BR), *3632* (BR, K), *4380* (BR), *4464* (BR, K), *4787* (BR, L), *5093* (BR), *5625* (BR), *5677* (BR), *5723* (BR), *5831* (BR, K), *6128* (BR, K) & *6133* (BR); *Fruth, Mabonso & Lubini 192* (BRLU, WAG); *Gérard 2140* (BR); *Gentil s.n.* (BR); *Germain 180* (BR, WAG) & *7436* (BR, WAG); *Gilbert 1875* (BR, WAG), *2353* (BR), *7917* (BR), *8183* (BR), *9205* (BR), *9938* (BR) & *10204* (BR, BR, EA, K, WAG); *Gillet s.n.* (BR), *s.n.* (BR), *339* (BR) & *2919* (BR); *Gillet & Oddon 3333* (BR, WAG); *Gillet & Van Houtte 3665* (BR); *Goossens 4171* (BR); *Gutzwiller 1343* (BR, WAG), *1757* (BR) & *3719* (BR); *Hart 497* (BR) & *1439* (BR); *Hendrickx 139* (BR); *Homber 61* (BR, WAG); *Hulstaert 1068* (BR); *Hutchinson & Gillett 4115* (K); *Ichikawa 150* (BR); *Jans 532* (BR); *Jespersen 27* (BR); *Léonard J.J.G. 231* (BR, WAG); *Léonard* *A*. *1771* (BR, WAG); *Laurent M.D.J. s.n.* (BR), *s.n.* (BR), *s.n.* (BR), *s.n.* (BR), *406* (BR) & *2004* (BR); *Laurent É. & Laurent M.D.J. s.n.* (BR), *s.n.* (BR) & *s.n.* (BR); *Lebrun 1019* (BR), *1283* (BR, K) & *2688* (BR, WAG); *Lejoly 3488* (BRLU, WAG), *4457* (BRLU, WAG), *7233* (BRLU, WAG) & *7262* (BRLU, WAG); *Lewalle 1779* (BR, MO); *Liben 2600* (BR, WAG); *Lisowski 15611* (BR, POZG), *16034* (POZG), *16071* (BR, POZG), *17098* (POZG), *24298* (POZG), *24299* (POZG), *24300* (POZG), *24301* (POZG), *24302* (BR, POZG), *40408* (POZG), *44357* (POZG), *48403* (POZG), *81913* (POZG) & *86299* (POZG); *Lisowski, Malaisse, Symoens 2179* (POZG), *4955* (POZG), *5078* (POZG), *5623* (POZG) & *11553* (BR); *Louis 83* (B, BR, K, P), *7533* (BR), *8239* (BR), *10703* (BR, NY, WAG), *10987* (BM, BR, NY, WAG), *11252* (BM, BR, K, P), *14223* (BR, K), *16312* (BR); *Luke 10503* (BR, EA, K, MO, WAG); *Malaisse 9527* (BR), *13330* (BR, WAG) & *13866* (BR, WAG); *Masens 365* (BR); *Mbambi 146* (BR); *Mestdagh 421* (BR); *Mortehan 43* (BR, WAG) & *713* (BR); *Mosango 605* (BR); *Nannan 67* (BM); *Pauwels 3699* (BR, EA, K, WAG), *4841* (BR, WAG), *4890* (BR) & *5585* (BR); *Pittery 226* (BR) & *533* (BR); *Pynaert 146* (BR) & *550* (BR); *Robyns 473* (BR, K, WAG); *Sapin s.n.* (BR) & *s.n.* (BR); *Schaijes 2306* (BR); *Schmitz 266* (BR, WAG) & *645* (BR); *Seret 397* (BR, WAG); *Spitaels 291* (BR); *Streel 279* (BR); *Symoens 304* (BRLU, WAG) & *12354* (B, BR, K, WAG); *Szafrański 1310* (BR); *Toussain 2334* (BR); *Troupin 14* (BR, K, WAG), *242* (BR) & *257* (BR, K, WAG); *Unreadable collector name 680* (BR); *Vanderyst 3549* (BR), *4346* (BR), *14414* (BR), *15359* (BR), *15634* (BR), *19174* (BR), *28934* (BR), *34511* (BR), *34607* (BR), *35062* (BR) & *35067* (BR);

**Republic of the Congo.** *Attims 46* (P); *Bitsindou 107* (BRLU, WAG); *Bouquet 481* (P); *Champluvier 5238* (BR); *Chevalier 5118* (P) & *10925* (P).; *Cusset 1246* (P); *de Foresta 985* (P) & *1574* (P); *de Namur 1798* (IRSC); *de Néré* 923 (P) *& 1716* (P); *Descoings 7697* (IEC, WAG); *Farron 4058* (P); *Hallé 1716* (IRSC); *Harris 9136* (BR); *Koechlin 826* (P); *Lisowski 7032* (BR); *Moutsamboté 6676* (K); *Moutsamboté, Pollard, Nsongola, Nzita, M'Boungou, Tsoumou & Weber 6633* (IEC, K, WAG); *N'Kounkou 115* (BR, WAG); *Ndolo Ebika 207* (E); *Sita 1476* (P), *1601* (P), *3200* (P) & *3894* (P, WAG); *Thollon s.n.* (P), *1222* (K, P, WAG) & *4074* (K, P, WAG); *Tisserant & Berget s.n.* (P) & *s.n.* (P).

**Equatorial Guinea.** *Eneme Efua & Lejoly 6* (BRLU, WAG); *Guinea 2488* (MA); *Lejoly 270* (BRLU, WAG) & *377* (BRLU, WAG); *Lisowski 1279* (BRLU, WAG); *Luke 13342* (EA, K, WAG); *Luke, Posa Bohome, Johansen & Lehman 11904* (EA, K, WAG); *Luke & Fermin 12115* (EA, K, MA, MO, WAG); *Ngomo & Ndong 380* (BRLU, WAG); *Parmentier & Esono 3071* (BRLU, WAG), *3372* (BRLU, WAG) & *3511* (BRLU, WAG); *Senterre & Ngomo s.n.* (BRLU), *s.n.* (BRLU), *s.n.* (BRLU) & *3560* (BRLU); *Senterre & Obiang Mbomio s.n.* (BRLU), *s.n.* (BRLU), *s.n.* (BRLU) & *s.n.* (BRLU).

**Gabon.** *Bidault, Sonké, Akouangou, Ikabanga & Boussiengui 1030* (LBV, MO, WAG); *Bissiengou, Nzabi, Ngombou Mamadou & Boulanga 1351* (LBV, WAG); *Bos 10421* (WAG); *Bos, van der Laan & Nzabi 10565* (WAG), *10602* (WAG), *10633* (BR, MO, WAG), *10751* (BR, MO, P, WAG), *10805* (WAG) & *10820* (BR, MO, P, WAG); *Boupoya-Mapikou, Lejoly & Moungazi 22* (BRLU, WAG); *Boupoya-Mapikou, Dauby & Nzabi 595* (BRLU, LBV, MO, WAG); *Boussiengui Nongo, Ngombou Mamadou, Ngoubou, Mouanga, & Moyiya118* (MO); *Breteler & deWilde J.J.F.E. 1* (WAG), *496* (WAG), *581* (WAG); *Breteler 6609* (WAG), *6765* (WAG), *7590* (WAG), *7609* (WAG) & *7678* (MO, WAG); *Breteler, Jongkind & Wieringa 11332* (LBV, WAG); *Breteler, Jongkind, Wieringa & Moussavou 9876* (LBV, MO, WAG) & *9997* (LBV, MO, WAG); *Breteler & Lemmens 8412* (BR, MA, MO, P, WAG); *Breteler, Lemmens & Nzabi 8227* (WAG) & *8250* (WAG); *Breteler & van Raalte 5661* (WAG); *Damen 676* (WAG); *Damen & Simons 491* (LBV, WAG); *Damen, Simons & Taylor 532* (BR, LBV, MO, WAG); *Damen, Simons & Niangadouma 616* (BR, LBV, WAG), 630 (LBV, WAG), 633 (LBV,WAG) & 663 (LBV, WAG); *de Wilde J.J.F.E. s.n.(*WAG); *de Wilde J.J.F.E., Arends, Louis, Karper, & Bouman 775* (BR, C, LBV ,MO, P, WAG); *Dhetchuvi Matchu-Mandje 1472* (BRLU, WAG) & *1565* (BRLU, WAG); *Doumenge 76* (LBV, P); *Florence 902* (P), *968* (P), *1522* (P, WAG), *1536* (P), *1625* (P) & *1902* (P); *Floret, Louis & Moungazi 1655* (P, WAG); *Hallé 1412* (P), *2339* (P) &3385 (P); *Hallé & Villiers 4395* (P), *5289* (P) & *5311* (P); *Harris, Armstrong & Niangadouma 8381* (E, LBV, MO); *Jeffrey 311* (K); *Klaine s.n.* (P)*, 118* (P), *278* (P, WAG), *466* (K,P) & *802* (P), *Koenen, Leal, Bissiemou, Mounoumoulossi & Nguema Ekomo 30* (LBV, WAG) & *56* (LBV, WAG); *Lachenaud, Walters, Boussiengui & Moungoudi 1144* (BR, LBV, MO); *Le Testu 1301* (P), *2001* (BR, K, P, WAG), *7898* (MO, P, WAG) & *9144* (P, WAG); *Leal, Nguema Ekomo, Mounoumoulossi & Bissiemou 1010* (MO, WAG), *502* (MO) & *975* (MO); *Lecomte 20* (K, P, WAG); *Leeuwenberg 11393* (WAG), *11460* (WAG), *11513* (WAG), *12487* (MO, WAG), *12535* (WAG) & *12552* (MO, WAG); *Louis 1547* (LBV) & *3011* (LBV, MO, WAG), 3055 (LBV, WAG); *Louis & Blom 2904* (LBV, MO, WAG); *Louis, Breteler & de Bruijn s.n.* (WAG), *216*(WAG)*, 220* (WAG)*, 929* (WAG) & *1008* (WAG); *Louis & Nzabi 2981* (LBV, WAG) & *2987* (LBV); *Maas, Breteler, Maas-van de Kamer, Niangadouma & Specht 10087* (LBV, MO, UC, WAG); *van der Maesen, Louis & de Bruijn 5542* (LBV, WAG); *Mayombo-Nzengue 298* (LBV, WAG); *McPherson 15834* (LBV, MO); *Minkébé Series 2* (WAG); *Minkébé Series & Wilks 506* (WAG) & *525* (WAG); *Moungazi* 1589 (LBV, MO, WAG); *Ngok Banak, Moungazi & Lekanga 1099* (BRLU, LBV, WAG); *Ngombou Mamadou, Boussiengui Nongo & Mouanga 99* (MO); *Nguema Ekomo, Mounoumoulossi Bissiemou & Dauby 950* (LBV); *Niangadouma & Stone 347* (BR, LBV, MO, WAG); *Parmentier & Nguema Miyono 611* (BRLU, WAG), *780* (BRLU, WAG) & *820* (BRLU, LBV); *Quiroz-Villarreal, Boogmans, Ruysschaert & van Andel 1143* (LBV, WAG); *Reitsma J.M. & Reitsma B. 2212* (WAG); *Reitsma J.M., Reitsma B. & Louis1803* (MA, MO, NY, WAG) & *2114* (MA, MO, NY, WAG); *Sita 539* (P), *5097* (LBV) & *5287* (LBV); *Sosef, Azizet Issembé, Bourobou & Moussavou 961* (BR, LBV, MO, WAG); *Sosef, Breteler, Azizet Issembé & Moussavou 1225* (LBV, MO, WAG) & *1346* (LBV, MO, WAG); *Sosef, Leal, White, Abernethy & Azizet Issembé 711* (LBV, WAG); *Soyaux 61* (GOET, K, Z); *Stévart, Azizet Issembé, Akouangou & Boussiengui 3962* (BR, LBV, MO, WAG); *Stévart, Dauby, Mounoumoulossi, Bissiemou & Akouangou 3397* (MO); *van der Laan 230* (WAG); *van Setten 694* (WAG); *van Valkenburg, Degreef, Azizet Issembé & Boussiengui 2990* (BR, K, LBV, MO, WAG); *Walker s.n.* (P) & *s.n.* (P, WAG); *Walters, Davidson, Christoph & van de Weghe 1667* (LBV, MO); *Wieringa 544* (LBV, MO, WAG), *650* (WAG) & *1272* (LBV, MO, WAG); *Wieringa, Hoekstra, Niangadouma & Boussiengui 6103* (LBV, WAG); *Wieringa, Jongkind, Schoonhoven & Mbombet 3896* (LBV, WAG); *Wieringa & van de Poll 1502* (WAG); *Wieringa, van Nek & Hedin 2882* (WAG); *Wieringa, van Proosdij & Nzabi 7720* (WAG);

**Ghana.** *Adams 2128* (GC, K) & *2266* (GC); Burton & Commerson s.n. (K); *Enti s.n.* (GC); *Hall s.n.* (GC) *& s.n.* (GC)*; Hall & Enti 38524* (GC, K); *Hall & Swaine 43236* (GC); *Jongkind 3754* (MO, WAG); *Schmidt, Merello, Amponsah & Welsing 2018* (MO, WAG); *Hall & Lock 46477* (GC); *Enti 725* (MO, NY, WAG) & *748* (MO, PRE); *Komla s.n.* (GC); *Plumptre 76* (K); *Polley s.n.* (GC); *Roberty 12785* (G); *Vanderpuije s.n.* (GC); *Vigne 4091* (K, P).

**Guinea.** *Adam 5968* (MO, P); *Bilivogui & Lerat 227* (MO, SERG); *Bosch, Haba & Keita 361* (BR, WAG); *Chevalier 12439* (P), *12490* (P), *12557* (P), *12658* (P), *12909* (P), *13216* (P) & *13616* (P); *Chevalier & Caille 14967* (K, P); *Haba, Burgt, van der Couch, Haba, Traoré et al. 93* (HNG, K); *Haba, Goman & Diallo 283* (HNG, K, WAG); *Jongkind, Bilivogui & Diabate 11562* (MO, WAG);

*Jongkind, Bilivogui, Koenen, Kortekaas & Mulbah 10730* (BR, MO, O, WAG); *Jongkind, Holié & Cherif 7766* (P, WAG); *Jongkind, Nema & Holié 7487* (WAG); *Lisowski 7352* (POZG); *Pobéguin 612* (P), *613* (P), *1429* (P) & *1922* (P); *Schnell 949* (IFAN, P, PRE), *2329* (IFAN), *4782* (P) & *5146* (P); *Scott-Elliot 4551* (A, BM, K) & *5009* (A, BM, K); *Traoré, Pearce, Fatoumata & Manimou 115* (HNG, K).

**Ivory Coast.** *Aké Assi 220* (UCJ), *5657* (UCJ), *5691* (UCJ), *6056* (UCJ), *8338* (UCJ), *8352* (UCJ), *8387* (K), *9242* (K) & *10131* (UCJ); *Antheunisse 40* (WAG), *49* (WAG), *50* (WAG), *145* (WAG), 146 (WAG) & *147* (WAG); *Bamps 2578* (BR); *Beentje 203* (UCJ, WAG), *231* (UCJ, WAG) & *412* (MO, UCJ, WAG); *Bos 10334* (WAG), *10337* (WAG), *10340* (WAG), *10361* (BR, MO, WAG), *10407* (WAG) & *10408* (WAG); Breteler 5202 (BR, MO, WAG), 6020 (WAG) & 7362 (WAG); *César 1174* (P) & *1512* (P); *Chevalier 19066* (P) & *19806* (P, WAG); *de Koning 1864* (BR, E, MO, O, WAG), *2606* (WAG), *2610* (BR, MO, O, WAG), *3157* (WAG), *3160* (WAG), *3253* (WAG), *3256* (WAG), *3586* (BR, C, E, FR, G, K, MA, MO, O, P, PRE, WAG), *3751* (WAG), *3753* (BR, E, MO, O, WAG), *4662* (BR, E, MO, WAG) & *5846* (BR, C, E, G, MA, MO, O, WAG); *de Namur 409* (ABI), *500* (ABI) & *850* (ABI, WAG); *Farron s.n.* (WAG) & *s.n.* (WAG); *Geerling & Bokdam 1536* (WAG); *Guillaumet 939* (ABI); *Hall & Abbiw 45497* (GC); *Hepper & Maley 7961* (K); *Jongkind & Assi-Yapo 5023* (WAG); *Jongkind, Hawthorne & Assi-Yapo 4666 (WAG); Jongkind & Students of the 'Université de Cocody' 4456* (WAG) & 4481 (BP, IAGB, MO, WAG); *Kouamé 1219* (CSRS), *1418* (CSRS) & *1611* (CSRS); *Leeuwenberg 2217 (K, WAG), 8108 (WAG), 12047* (ABI, WAG) & *12309* (WAG); *Nusbaumer 653* (G, P); *Oldeman 984* (BR, K, WAG); *de Rouw 191* (WAG) & *294* (WAG); *Schnell 3922* (P); *Stäuble 458* (G); *Thijssen 160* (MO, WAG); *van der Burg 289* (MO, WAG), *818* (WAG) & *1023* (WAG); *van Doorn & van Doorn-Hoekman 33* (BR, MO, WAG).

**Liberia.** *Adam 16654* (MO), *21420* (K), *25282* (MO), *26275* (MO) & *29737* (MO, P); *Baldwin jr 6178* (K) & *7012* (K); *Daniels, Dorbor & Sambolah 64* (BR, MO, SL, WAG); *Harley 456* (B); *Jansen 1108* (WAG); *Jongkind & Bilivogui 9654* (BR, MO, WAG); *Jongkind, Bilivogui & Dorbor 8871* (BR, MO, WAG); *Jongkind & Blyden et al. 5293* (WAG); *Jongkind, Daniels Konie & Gorpudolo 7203* (WAG); *Jongkind, Kwewon, Kpadeyeah, Konie, Morgan & Daniels 6347* (WAG); *Jongkind & Sambolah 13093* (WAG); *Linder 765* (K); *Senterre & Weah 6955* (BRLU, MO, WAG).

**Nigeria.** *Bos 10427* (WAG); *Brenan 8599* (K) & *9045* (B, K); *Brenan, Onochie, Jones & Richards 9073* (K); *Daramola 62736* (K, P, WAG) & *72332* (FH, FHI, K, MO); *Ekwuno, Adetula & Oguntayo 67016* (FHI); *Ekwuno, Fagbemi & Ihe 76* (FHI); *Emwiogbon 57515* (FHI), *57949* (FHI), *60034* (FHI), *60660* (FHI), *61140* (FHI), *61702* (FHI) & *66568* (FHI); *Kennedy 1135* (K, S, WAG), *1783* (K) & *2645* (K); *Leeuwenberg 11258* (WAG), *11264* (BR, C, E, FHO, FR, MA, MO, O, PRE, WAG) & *11318* (MO, WAG); *Lowe 914* (UCI) & *2043* (FHI, K,UCI); *Ntui 724* (MO); *Odewo & Adedeji 258* (FHI); *Okafor & Emwiogbon 66113* (FHI, K, UCI); *Olorunfemi, Jbinuyo & Babagbemi 493* (FHI, WAG); *Onochie 27700* (FHI), *31243* (FHI, K), *34299* (FHI) & *35693* (FHI); *Richards 3309* (BM); *Ross 253* (BM); *Sijnade 510* (IFE); *Talbot 730* (BM, K), *3032* (BM) & *3667* (BM); *van Meer 1661* (FHI, WAG); *Wit & Gbile 974* (WAG).

**Sierra Leone.** *Thomas 4169* (K).

**Tanzania.** *Fromm & Münzner 245* (B, WAG) & *261* (B).

**Zambia.** *Angus 406* (K) & *409* (BM, BR, FHO, K, PRE); *Breton 5307* (BR); *Bullock 1106* (BR, K,US) & *3862* (BR, EA, K, MO); *Congdon 697* (K); *Eyles 8217* (BM, K, SRGH); *Fanshawe 1021* (BR, FHO, K, SRGH), *1367* (BR, FHO, K), *3207* (K), *3543* (K), *8207* (K) & *8624* (K); *Fries 439* (UPS); *Goyder, Pope & Radcliffe-Smith 3128* (K); *Harder, Schmidt & Bolnick 3003* (MO, WAG); *Hooper & Townsend 35* (K); *Lawton 990* (FHO); *Mutimushi 488* (FHO) & *3033* (K, NDO); *Richards 15266* (K, SRGH), *1845* (BR, EA, K) & *5561* (K); *Saint-Clair Thompson 1094* (K); *Sanane 87* (B, K); *Schmitz 3491* (BR, FHO); *Whellan 1552* (SRGH); *White 3405* (BR, FHO, K, PRE).

Table S2.3. List of validated herbarium records of *Dracaena surculosa* by country of origin. Structure of the table: Collector(s) name, collector number, owning herbarium(a)

**Benin.** *Adjakidjč 4690* (BENIN, WAG); *Adjanohoun 74* (K, P); *Akočgninou 3313* (BENIN, BR, MO, WAG); *Akočgninou, Agbani & Yédomonhan 2150* (BENIN, BR, MO, WAG) & *2208* (BENIN, MO, WAG); *Chevalier 23015* (P, WAG); *Lisowski 174* (BENIN, POZG, WAG); *Pauwels 8300* (BENIN, BRLU, MO, WAG); *Sokpon 2100 (BENIN, WAG); Sokpon, Yédomonhan & Trepo 1095* (BENIN, WAG); *van der Maesen, Akočgninou, Essou & Agbani 6620* (BENIN, MO, WAG); *van der Maesen, Akočgninou, Essou & Sokpon 6295* (BENIN, WAG) & *6340* (BENIN, WAG).

**Cameroon.** *Letouzey 13701* (K, P, WAG, YA); *Onana 53* (P, WAG, YA); *Sainge & Ekpe 30* (MO, YA); *Winkler 491* (B, WAG, Z).

**Ghana.** *Brown 370* (K); *Chipp 570* (K); *Dalziel 8292* (K); *de Wit & Morton 2932* (B, GC, K, WAG); *Enti & Hall 37502* (K); *Hall 2829* (K); *Hall & Enti 36310* (K); *Hall & Jenik 3596* (K); *Hall & Swaine 46552* (GC, K, WAG); *Harder, Schmidt, Amponsah & Kipo 2915* (GC, MO, WAG); ; *Irvine 1992* (K), *2307* (GC), *3207* (K) & *4787* (K); *Johnson 628* (K); *Jongkind 2350* (BR, MO, WAG); *Jongkind, Abbiw, & Markwei 1650* (MO, WAG); *Kinloch 3240* (K, P); *Leeuwenberg 11100* (GC, WAG), *11117* (GC, WAG), *11909* (BR, GC, HUJ, MO, O, WAG), *11966* (GC, WAG) & *12008* (GC, WAG); *Lock 46725* (K); *Lyon 2865* (K); *Morton 6143* (GC, K); *Veldkamp 6129* (K, L, WAG); *Vigne FH1318* (K) & *FH2061* (K);

**Guinea.** *Adam 30379* (MO, P, SRGH), *3754* (P) & *4680* (MO, P); *Bilivogui, Diabate, McPherson & Serein 21* (MO, P, SERG); *Bilivogui & Jongkind 178* (BR, MO, P, SERG, WAG); *Cheek, Diabate & Guilavogui 13885* (HNG, K); *Chevalier 13311* (P) & *13344* (P); *Diabate, Bidault & Mas 1190* (MO, P, SERG, WAG); *Haba & Tchiengué 69* (K, WAG); *Jacques-Félix s.n.* (P) & *955* (P, WAG); *Jaeger 92* (K, P); *Jongkind & Bilivogui 11328* (MO, WAG); *Jongkind, Holié & Cherif 7718* (WAG); *Lisowski 7673* (POZG); *Mas, Diabate & Bidault 1194* (MO, SERG, WAG) & *1206* (MO, P, SERG, WAG); *Pobéguin 1132* (P); *Schnell 837* (P) & *1437* (P); *Scott-Elliot 4815* (K); *van der Burg, Haba & Goman 1267* (HNG, K, WAG).

**Ivory Coast.** *Aké Assi 8381* (K); *Beentje 406* (MO, UCJ, WAG), *451* (MO, UCJ, WAG), *813* (WAG) & *1089* (BR, MO, UCJ, WAG), *1121* (UCJ, WAG), *1354* (AMD, BR, MO, WAG); *Bernardi 8184* (P, WAG), *8516* (K, P) & *8692* (P); *Bos 1775* (WAG), *7354* (WAG), *7456* (WAG), *10265* (WAG), *10349* (WAG), *10352* (WAG), *10354* (WAG), *10366* (WAG), *10367* (WAG), *10396* (WAG) & *10428* (WAG); *Boughey 18217* (K);. *Bouquet & Debray 668* (G, WAG); *Breteler 5212* (BR, MO, P, WAG), *5820* (BR, WAG), *5848* (BR, K, MO, P, PRE, WAG), *7346* (WAG), *7476* (BR, MO, WAG), *7481* (WAG) & *13752* (WAG); *Chevalier s.n.* (P), *s.n.* (P), *15203* (P), *15487* (P), *16768* (P), *16768* (P), *16782* (P), *16824* (P), *16833* (P), *17056* (P), *19065* (P), *19142* (P), *19275* (P), *21523* (P), *21589* (P); *de Kruif 210* (UCJ, WAG); *de Rouw 120* (WAG) & *252* (WAG); *de Wilde J.J.F.E. 279* (WAG), *317* (WAG), *335* (WAG), *672* (WAG); *de Wilde J.J.F.E. & Leeuwenberg 3433* (BR, K, P, WAG); *de Wilde W.J.J.O. 202* (WAG) & *1091* (B, BR, EA, K, P, SRGH, WAG, Z); *de Wit 124* (WAG) & *8141* (WAG); *Farron s.n.* (WAG), *s.n.* (WAG), *s.n.* (WAG), *s.n.* (WAG); *Gautier, Kouamé, Bänninger 1812* (G); *Gautier, Kouamé, Etien & Téré 51387* (G, WAG); *Giovannetti 121* (IFAN); *HallĂ© 268* (P); *Hepper & Maley 7819* (K, P) & *8066* (K, P); *Jangoux 406* (BRLU, WAG) & *479* (BR, BRLU); *Jongkind & Assi-Yapo 4932* (WAG); *Jongkind, Hawthorne & Assi-Yapo 4667* (MO, WAG); *Jongkind, Hawthorne, Assi-Yapo & Aman 4744* (WAG); *Jongkind & Mertens 12816* (BR, WAG); *de Koning 86* (MO, WAG), *1107* (BR, E, G, MA, MO, O, WAG), *1152* (BR, E, MA, MO, O, WAG), *1155* (BR, E, MA, MO, O, WAG), *1166* (BR, MO, WAG), *1310* (BR, E, MO, WAG), *1319* (WAG), *1403* (WAG), *1557* (BR, E, MO, WAG), *1571* (BR, E, MA, MO, O, WAG), *1602* (BR, E, MO, O, WAG), *1617* (BR, C, E, G, MA, MO, O, WAG), *1645* (BR,E,MO,WAG), *1753* (BR, E, MO, WAG), *1776* (BR, MO, WAG), *2112* (WAG), *2220* (BR, E, G, MA, MO, O, WAG), *2256* (WAG), *2467* (BR, E, MO, WAG), *2569* (WAG), *2595* (BR, MO, WAG), *3034* (MO, WAG), *3348* (WAG), *3547* (WAG), *3673* (WAG), *3788* (WAG), *5394* (BR, E, MO, O, WAG), *5395* (MO, WAG) & *5683* (BR, E, MA, MO, O, WAG); *Kouamé, Bänninger, Gautier &, Chatelain 569* (G); *Kouamé, Téré & Barriera 158* (G); *Leeuwenberg 2262* (WAG), *2362* (K, WAG), *2593* (WAG), *2678* (WAG), *2894* (WAG), *7921* (BR, FHI, HBG, LISC, MO, P, PRE, UPS, WAG), *7937* (BR, MO, WAG), *7943* (BR, MO, WAG), *8109* (BR, FHI, HBG, K, LISC, MO, P, PRE, UPS, WAG), *8111* (P, WAG), *12164* (WAG), *12282* (WAG); *Thijssen 16* (WAG), *195* (WAG), *213* (WAG) & *309* (BR, FR, HUJ, IFAN, K, MO, O, POZG, WAG); *van der Burg 114* (MO, WAG), *161* (WAG), *162* (WAG), *264* (WAG), *265* (WAG), *266* (WAG), *288* (MO, WAG), *418* (WAG), *701* (WAG); *van Doorn & van Doorn-Hoekman 18* (BR, MO, WAG), *74* (BR, HUJ, K, MO, O, POZG, WAG) & *86* (MO, WAG); *van Setten 314* (WAG); *Wieringa 4301* (WAG); *Wieringa, Assi-Yapo* & *Mabea 4268* (BR, MO, WAG).

**Liberia.** *Adam 16196* (P) & *27683* (MO, P, PRE, WAG); *Baldwin 6135* (K), *6310* (K), *10380* (K) & *11252* (K); *Blyden 69* (BR, MO, WAG); *Bos 2215* (BR, K, LIB, P, WAG); *Cook 295* (US); *Dinklage 2654* (B); *Harley 906* (B) & *1210* (WAG); *Jansen 1529* (BR, MO, WAG) & *1718* (WAG); *Jongkind, Bilivogui & Daniels 10146* (WAG); *Jongkind, Bilivogui & Dorbor 9396* (WAG); *Jongkind, Daniels, Konie & Gorpudolo 7217* (BR, G, K, WAG); *Jongkind, Kwewon, Kpadeyeah, Konie, Bafaie & Daniels 6082* (WAG); *Jongkind, Kwewon, Kpadeyeah, Konie, Morgan & Daniels 6390* (WAG); *Jongkind & Sambolah 13038* (WAG); *Leeuwenberg & Voorhoeve 4587* (B, K, WAG); *Linder 74* (K, MO ) & *1025* (K, MO); *Stoop - v.d. Kasteele 244* (WAG), *310* (MO, WAG) & *341* (WAG); *Straub 839* (US); *van Meer 103* (MO, WAG); *Voorhoeve 776* (K, WAG); *Whyte s.n.* (K, P), *s.n.* (K), *s.n.* (K) & *s.n.* (K), *s.n.* (P),; *Yonon Botanic Team 15* (WAG).

**Nigeria.** *Barter 2095* (K); *Binuyo 45464* (FHI, K, MO); *Brenan, Jones, Onochie & Richards 9027* (B, BR, K, P, WAG); *Brown & Opayemi 998* (DES, MO, NY, WAG); *Chapman 4250* (K); *Coombe 131* (K); *Dodd 374* (K, P, Z); *Fagbemi 24* (FHI); *Gledhill 769* (FHI, K, P, WAG); *Hall 1583* (K);

*Latilo, M.G. & Daramola, B.O 28822 (K);* *Latilo 133* (K); *Leeuwenberg 11330* (BR, E, MO, WAG); *Mann 2327* (K, P, WAG); *Meikle 782* (B, K, P, WAG); *Millen 144* (K); *Odewo & Adedeji 99942* (FHI, WAG); *Oguntayo & Adejimi 218* (FHI); *Okafor & Daramola 35293* (K); *Olorunfemi 55661* (K); *Pilz 2533* (B, MO); *Sharland 1066* (K); *Talbot 146* (K) & 1421 (BM, K); *van Meer 601* (WAG), *883* (WAG) & *1691* (WAG); *Wit & Daramola 1048* (WAG).

**Sierra Leone**. *Afzelius s.n.* (BM, WAG); *Dalziel 947* (C, E, K, Z); *Dawe 440* (K); *Deighton 1245* (K), *3859* (K) & *5652* (K); *Haswell 132* (K); *Jaeger 340* (P), *1228* (P), *1466* (P), *4131* (P), *6871* (P) & *7135* (P); *Jordan 2062* (K); *Milne-Redhead 5190* (K); *Morton 1622* (FHI, GC, IFAN, K, SL, WAG) & *1773* (FHI, GC, K, SL, WAG): *Morton & Jarr 2202* (K, MO, SL, WAG) & *3355* (FHI, GC, IFAN, K, SL, WAG); *Small 582* (K); *Thomas 1813* (K), *2827* (K), *2990* (K), *7477* (K).

**Togo.** *Brunel 155* (B); *Ern 2867 (B, K); Hakki, Leuenberger & Schiers 602* (B); *Mahoux 79* (P, WAG).

Appendix 3

| Mean annual temperature | 1 | 1 | 0.03 | 0.14 | -0.15 | 0.65 | 0.69 | -0.08 | 0.76 | 0.57 | 0.76 | 0.80 | -0.02 | 0.07 | -0.07 | 0.46 | 0.03 | -0.07 | -0.16 | 0.08 | yes |
| --- | --- | --- | --- | --- | --- | --- | --- | --- | --- | --- | --- | --- | --- | --- | --- | --- | --- | --- | --- | --- | --- |
| Mean diurnal temperature range | 2 | 0.03 | 1 | -0.50 | 0.59 | 0.58 | -0.59 | 0.81 | 0.12 | -0.17 | 0.39 | -0.35 | -0.63 | -0.52 | -0.45 | 0.29 | -0.52 | -0.50 | -0.56 | -0.42 | no |
| Isothermality | 3 | 0.14 | -0.50 | 1 | -0.92 | -0.56 | 0.70 | -0.88 | 0.14 | -0.01 | -0.48 | 0.65 | 0.74 | 0.65 | 0.53 | -0.08 | 0.64 | 0.58 | 0.63 | 0.43 | no |
| Temperature seasonality | 4 | -0.15 | 0.59 | -0.92 | 1 | 0.61 | -0.74 | 0.94 | -0.14 | 0.03 | 0.52 | -0.71 | -0.77 | -0.77 | -0.37 | -0.06 | -0.75 | -0.42 | -0.63 | -0.40 | yes |
| MAX temperature of warmest_month | 5 | 0.65 | 0.58 | -0.56 | 0.61 | 1 | -0.04 | 0.68 | 0.44 | 0.49 | 0.97 | 0.10 | -0.58 | -0.48 | -0.37 | 0.34 | -0.50 | -0.40 | -0.64 | -0.22 | no |
| MIN temperature of the coldest month | 6 | 0.69 | -0.59 | 0.70 | -0.74 | -0.04 | 1 | -0.76 | 0.49 | 0.45 | 0.12 | 0.95 | 0.54 | 0.54 | 0.32 | 0.17 | 0.51 | 0.36 | 0.35 | 0.43 | No |
| Annual temperature range | 7 | -0.08 | 0.81 | -0.88 | 0.94 | 0.68 | -0.76 | 1 | -0.07 | -0.01 | 0.54 | -0.63 | -0.78 | -0.71 | -0.47 | 0.09 | -0.70 | -0.53 | -0.68 | -0.45 | Yes |
| Mean temperature of wettest quarter | 8 | 0.76 | 0.12 | 0.14 | -0.14 | 0.44 | 0.49 | -0.07 | 1 | 0.08 | 0.52 | 0.60 | -0.06 | 0.02 | -0.06 | 0.55 | -0.01 | -0.07 | -0.03 | -0.07 | No |
| Mean temperature of driest quarter | 9 | 0.57 | -0.17 | -0.01 | 0.03 | 0.49 | 0.45 | -0.01 | 0.08 | 1 | 0.56 | 0.42 | 0.01 | 0.02 | 0.02 | 0.07 | -0.01 | 0.04 | -0.20 | 0.21 | No |
| Mean temperature of warmest quarter | 10 | 0.76 | 0.39 | -0.48 | 0.52 | 0.97 | 0.12 | 0.54 | 0.52 | 0.56 | 1 | 0.23 | -0.51 | -0.42 | -0.29 | 0.33 | -0.45 | -0.32 | -0.56 | -0.16 | Yes |
| Mean temperature of coldest quarter | 11 | 0.80 | -0.35 | 0.65 | -0.71 | 0.10 | 0.95 | -0.63 | 0.60 | 0.42 | 0.23 | 1 | 0.46 | 0.52 | 0.18 | 0.34 | 0.49 | 0.21 | 0.26 | 0.32 | Yes |
| Annual precipitation totals | 12 | -0.02 | -0.63 | 0.74 | -0.77 | -0.58 | 0.54 | -0.78 | -0.06 | 0.01 | -0.51 | 0.46 | 1 | 0.92 | 0.57 | -0.25 | 0.93 | 0.62 | 0.80 | 0.62 | Yes |
| Precipitation in the wettest month | 13 | 0.07 | -0.52 | 0.65 | -0.77 | -0.48 | 0.54 | -0.71 | 0.02 | 0.02 | -0.42 | 0.52 | 0.92 | 1 | 0.33 | 0.00 | 0.99 | 0.37 | 0.71 | 0.53 | Yes |
| Precipitation in the driest month | 14 | -0.07 | -0.45 | 0.53 | -0.37 | -0.37 | 0.32 | -0.47 | -0.06 | 0.02 | -0.29 | 0.18 | 0.57 | 0.33 | 1 | -0.41 | 0.34 | 0.98 | 0.52 | 0.42 | Yes |
| Precipitation seasonality | 15 | 0.46 | 0.29 | -0.08 | -0.06 | 0.34 | 0.17 | 0.09 | 0.55 | 0.07 | 0.33 | 0.34 | -0.25 | 0.00 | -0.41 | 1 | -0.04 | -0.44 | -0.22 | -0.24 | No |
| Precipitation in the wettest quarter | 16 | 0.03 | -0.52 | 0.64 | -0.75 | -0.50 | 0.51 | -0.70 | -0.01 | -0.01 | -0.45 | 0.49 | 0.93 | 0.99 | 0.34 | -0.04 | 1 | 0.39 | 0.73 | 0.54 | No |
| Precipitation in the driest quarter | 17 | -0.07 | -0.50 | 0.58 | -0.42 | -0.40 | 0.36 | -0.53 | -0.07 | 0.04 | -0.32 | 0.21 | 0.62 | 0.37 | 0.98 | -0.44 | 0.39 | 1 | 0.56 | 0.46 | No |
| Precipitation in the warmest quarter | 18 | -0.16 | -0.56 | 0.63 | -0.63 | -0.64 | 0.35 | -0.68 | -0.03 | -0.20 | -0.56 | 0.26 | 0.80 | 0.71 | 0.52 | -0.22 | 0.73 | 0.56 | 1 | 0.25 | No |
| Precipitation in the coldest quarter | 19 | 0.08 | -0.42 | 0.43 | -0.40 | -0.22 | 0.43 | -0.45 | -0.07 | 0.21 | -0.16 | 0.32 | 0.62 | 0.53 | 0.42 | -0.24 | 0.54 | 0.46 | 0.25 | 1 | No |
| Variable name | Variable ID | 1 | 2 | 3 | 4 | 5 | 6 | 7 | 8 | 9 | 10 | 11 | 12 | 13 | 14 | 15 | 16 | 17 | 18 | 19 | Remove variable |

Highlighted coefficients indicate values equal or higher than r=(±)0.75 being a threshold for the removal of redundant variable

Table S3.1. Testing of variable multicollinearity over study area using pairwise Pearson correlation coefficient and indication for removal of redundant variables.

Figure S3.1. Maximum training and test sensitivity + specificity logistic threshold for three *Dracaena* sp. Maxent models calibrated in this study

Table S3.2. Comparison of possible habitats with occurrence records of *Dracaena afromontana*

| Country | No. of validated location records with coordinates (record density per 10,000 km^2^) | No. of records finally used for SDM (record density per 10,000 km^2^) |
| --- | --- | --- |
| DR Congo | 25 (0.11) | 14 (0.06) |
| Ethiopia | 35 (0.31) | 27 (0.24) |
| Tanzania | 42 (0.45) | 33 (0.35) |
| Kenya | 23 (0.39) | 22 (0.37) |
| Rwanda | 5 (1.98) | 5 (1.98) |
| Uganda | 11 (0.46) | 7 (0.29) |
| Burundi | 13 (5.14) | 9 (3.31) |
| Malawi | 4 (0.34) | 2 (0.17) |
| South Sudan | 3 (0.05) | 3 (0.05) |

Table S3.2. Comparison of possible habitats with occurrence records of *Dracaena camerooniana*

| Country | No. of validated location records with coordinates (record density per 10,000 km^2^) | No. of records finally used for SDM (record density per 10,000 km^2^) |
| --- | --- | --- |
| Cameroon | 185 (3.98) | 137 (2.95) |
| Gabon | 129 (4.93) | 105 (4.01) |
| Nigeria | 39 (0.43) | 21 (0.23) |
| Equatorial Guinea | 20 (7.46) | 17 (6.34) |
| Ivory Coast | 73 (2.28) | 52 (1.62) |
| Liberia | 18 (1.88) | 17 (1.77) |
| Ghana | 19 (0.80) | 18 (0.75) |
| Congo DRC | 179 (0.77) | 124 (0.53) |
| Sierra Leone | 1 (0.14) | 1 (0.14) |
| Tanzania | 2 (0.02) | 2 (0.02) |
| Congo | 27 (0.79) | 27 (0.79) |
| Guinea | 29 (1.19) | 24 (0.98) |
| Angola | 5 (0.04) | 5 (0.04) |
| Zambia | 28 (0.37) | 24 (0.32) |
| Central African Republic | 16 (0.26) | 13 (0.21) |
| Burundi | 1 (0.37) | 1 (0.37) |

Table S3.3. Comparison of possible habitats with occurrence records of *Dracaena surculosa*

| Country | No. of validated location records with coordinates (record density per 10,000 km^2^) | No. of records finally used for SDM (record density per 10,000 km^2^) |
| --- | --- | --- |
| Ivory Coast | 135 (4.2) | 72 (2.3) |
| Liberia | 33 (3.4) | 27 (2.8) |
| Cameroon | 4 (0.1) | 4 (0.1) |
| Ghana | 29 (1.3) | 29 (1.3) |
| Guinea | 22 (0.9) | 19 (0.8) |
| Sierra Leone | 22 (3.0) | 16 (2.2) |
| Nigeria | 32 (0.4) | 28 (0.3) |
| Benin | 13 (1.2) | 11 (1.0) |
| Togo | 4 (0.4) | 4 (0.4) |

Figure S3.2. Changes in median *Dracaena* species range according to two contrasting climate change scenarios
